# In creatinine kinetics, the glomerular filtration rate always moves the serum creatinine in the opposite direction

**DOI:** 10.14814/phy2.14957

**Published:** 2021-08-17

**Authors:** Sheldon Chen, Robert Chiaramonte

**Affiliations:** ^1^ Section of Nephrology MD Anderson Cancer Center Houston TX USA; ^2^ Internal Medicine The State University of New York Downstate Health Sciences University Brooklyn NY USA

**Keywords:** creatinine clearance, differential equation, kinetic GFR, partial derivative

## Abstract

**Introduction:**

When the serum [creatinine] is changing, creatinine kinetics can still gauge the kidney function, and knowing the kinetic glomerular filtration rate (GFR) helps doctors take care of patients with renal failure. We wondered how the serum [creatinine] would respond if the kinetic GFR were tweaked. In every scenario, if the kinetic GFR decreased, the [creatinine] would increase, and vice versa. This opposing relationship was hypothesized to be universal.

**Methods:**

Serum [creatinine] and kinetic GFR, along with other parameters, are described by a differential equation. We differentiated [creatinine] with respect to kinetic GFR to test if the two variables would change oppositely of each other, throughout the gamut of all allowable clinical values. To remove the discontinuities in the derivative, limits were solved.

**Results:**

The derivative and its limits were comprehensively analyzed and proved to have a sign that is always negative, meaning that [creatinine] and kinetic GFR must indeed move in opposite directions. The derivative is bigger in absolute value at the higher end of the [creatinine] scale, where a small drop in the kinetic GFR can cause the [creatinine] to shoot upward, making acute kidney injury similar to chronic kidney disease in that regard.

**Conclusions:**

All else being equal, a change in the kinetic GFR obligates the [creatinine] to change in the opposite direction. This does not negate the fact that an increasing [creatinine] can be compatible with a rising kinetic GFR, due to differences in how the time variable is treated.

## Graphical abstract

Changes in creatinine are tightly coupled to changes in kinetic GFR. From an established differential equation, the derivative of creatinine with respect to kinetic GFR was calculated. With it, we mathematically prove that all else being equal, if the kinetic GFR goes down, then the creatinine must go up, and vice versa. The two always move in opposite directions.

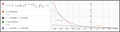

## 1 INTRODUCTION

Throughout the world, the serum creatinine is the most common measure for doctors to assess a patient's kidney function. Instinctively, they know that when the creatinine increases, the kidney function decreases (with exceptions), and vice versa. How quickly the creatinine is rising also has some bearing upon the severity of kidney function loss (Chiou & Hsu, 1975; Jelliffe, 2002; Mellas, 2016; Moran & Myers, 1985; Yashiro et al., 2012). Generally, the steeper the rise, the greater the impairment in the renal clearance. This creatinine slope is usually estimated from a graph generated by the electronic health record, and translating slope into kidney function is a decidedly qualitative art. Creatinine slope analysis became more quantitative when the science of creatinine kinetics was applied, a technique that has been called the kinetic glomerular filtration rate (GFR) (Chen, 2013, 2018a, 2018b; Chen & Chiaramonte, 2019). Now, the creatinine trajectory with all its slopes can be decoded into a kinetic GFR format that shows how the kidney function is evolving. Insight into the underlying kidney function by using the kinetic GFR has improved the diagnosis and treatment of patients who suffer from an acute kidney injury (AKI) (Bairy, 2020; Bairy et al., 2018; Dash et al., 2020; Dewitte et al., 2015; Endre et al., 2016; Khayat et al., 2019; Kwong et al., 2019; Latha et al., 2020; O'Sullivan & Doyle, 2017; Pianta et al., 2015; Yoshida et al., 2019). With creatinine kinetics being modeled mathematically, a question that naturally arises is how much does the [creatinine] change in response to a change in kinetic GFR? The answer can be found using derivatives.

### 1.1 Kinetic GFR equation

Changes in the serum creatinine follow passively from changes in the GFR, so like cause and effect, the change in GFR precedes and actively drives the change in creatinine. The creatinine serves mostly as a surrogate to calculate the GFR. The [creatinine] is relatable to the GFR by a differential equation (Chen, 2018a; Chen & Chiaramonte, 2019). The rate of change in the creatinine *amount* is a function of the rate of creatinine coming in minus the rate of creatinine going out of its volume of distribution, which is total body water (TBW) (Chow, 1985; Edwards, 1959; Jones & Burnett, 1974; Pickering et al., 2013). Most of the gain comes from creatinine being generated by the muscles, the mass of which remains more or less the same in the short term, so that the rate of creatinine addition is usually taken to be a constant: *Gen*. Most of the creatinine loss occurs via the kidneys, so that the rate of creatinine excretion is *GFR_K_* ·[*Cr*]*_t_*, a product of the kinetic GFR and the serum [creatinine] at an instant in time. On the left side of the differential equation, the instantaneous rate of change in creatinine amount is $\frac{d}{dt}\left({\left[Cr\right]}_{t}\cdot V_{t}\right)$, where [*Cr*]*_t_* is the serum [creatinine] at a given point in time, as before, and *V_t_* is the volume of distribution at the same point in time. Further, *V_t_* is allowed to vary at a steady rate to mimic the clinical reality that patients have many fluid inputs and outputs (I's/O's), like intravenous (IV) fluids and urine output. The net balance of the I's/O's divided by the time period over which they occur can be modeled as a constant rate of change in the creatinine's volume of distribution: $\frac{\Delta V}{\Delta t}$. Thus, volume as a function of time is $V_{t}=V_{0}+\frac{\Delta V}{\Delta t}t$, where *V*_0_ is the initial volume at a time zero for each clinical time interval. The full differential equation can be written as:

(1) $$\frac{d}{dt}\left({\left[\mathrm{Cr}\right]}_{t}\cdot V_{t}\right)=Gen‐GFR_{K}\cdot {\left[\mathrm{Cr}\right]}_{t}.$$

This first‐order linear differential equation's solution, as previously published, is:

(2) $${\left[\mathrm{Cr}\right]}_{t}={\left[\mathrm{Cr}\right]}_{0}+\underset{\text{Time evolution}}{\underbrace{\left[1‐{\left(\frac{V_{0}}{V_{0}+\frac{\Delta V}{\Delta t}t}\right)}^{\left(1+\frac{GFR_{K}}{\frac{\Delta V}{\Delta t}}\right)}\right]}}\cdot \underset{\left[\mathrm{Cr}\right]\;\text{spread}}{\underbrace{\left(\frac{\mathrm{Gen}}{GFR_{K}+\frac{\Delta V}{\Delta t}}‐{\left[\mathrm{Cr}\right]}_{0}\right)}}.$$

where ${\left[Cr\right]}_{0}$ is the initial serum [creatinine] for each clinical time interval (Chen, 2018a; Chen & Chiaramonte, 2019). That is to say, the serum [creatinine] at a given time is equal to the initial [creatinine] plus a time‐evolved portion of the spread between the initial [creatinine] and the [creatinine] reached at a new steady state if the kinetic GFR and volume change rate remained at those levels.

**FIGURE 1:**
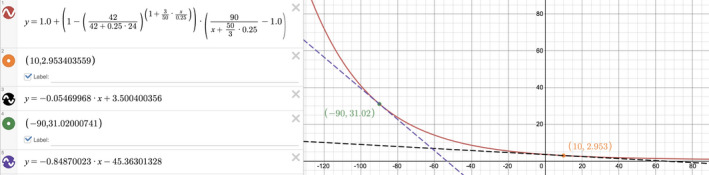
Visualizing the accuracy of $\frac{\partial {\left[\mathrm{Cr}\right]}_{t}}{\partial GFR_{K}}$ as written in Equation (3). The kinetic GFR Equation (2) is graphed as $GFR_{K}$ on the x‐axis and ${\left[\mathrm{Cr}\right]}_{t}$ on the y‐axis. The red curve slopes downward from left to right, and all of its tangent slopes appear to be negative. Two such tangent slopes at $GFR_{K}=10$ (orange dot) and $GFR_{K}=‐90$ (green dot) were calculated by the $\frac{\partial {\left[\mathrm{Cr}\right]}_{t}}{\partial GFR_{K}}$ equation. The derivative slopes are negative, as expected, and the calculated lines (black dash for $GFR_{K}=10$ and purple dash for $GFR_{K}=‐90$) do appear to be tangents.

To show how the [creatinine] trajectory is shaped by the kinetic GFR and other variables, we can graph the [*Cr*]*_t_* for a typical occurrence of AKI. Let us say that a patient with a baseline [creatinine] of 1.0 mg/dL develops acute tubular injury, and his GFR drops to 10 mL/min and stays there. He receives copious amounts of IV fluids in an attempt to reverse the renal failure, so that the net of the I's/O's is +6 L in 24 h, or $\frac{\Delta V}{\Delta t}=0.25\;L/h$. The creatinine generation rate is found by multiplying the baseline [creatinine] by its corresponding steady‐state GFR: Gen=1mg/dL×90mL/min. His initial volume of distribution, $V_{0}$, is taken to be TBW (Bjornsson, 1979), and a typical value is 42 L. The drop in GFR to a constant (average) value of 10 mL/min perturbs the steady state, so [creatinine] will change. We graphed the serum [creatinine] $\left(y\right)$ versus kinetic GFR $\left(x\right)$ to directly visualize the relationship between the two. If we imagine the tangent to this curve, it appears that the slope is consistently negative (Figure 1). We hypothesize that the tangential slope of [creatinine] versus kinetic GFR will always be negative.

## 2 MATERIALS AND METHODS

### 2.1 Derivative of [creatinine] with respect to kinetic GFR

From kinetic GFR Equation (2), we can differentiate the serum [creatinine], ${\left[\mathrm{Cr}\right]}_{t}$, with respect to the kinetic GFR, $GFR_{K}$, to find the instantaneous relationship between the two, assuming that all of the other variables are constant. This derivative $\left(\frac{\partial {\left[\mathrm{Cr}\right]}_{t}}{\partial GFR_{K}}\right)$ allows us to calculate the slope of the tangent to the curve of ${\left[\mathrm{Cr}\right]}_{t}$ versus $GFR_{K}$. In Equation (2), the derivative of $\left[1‐{\left(\frac{V_{0}}{V_{0}+\frac{\Delta V}{\Delta t}t}\right)}^{\left(1+\frac{GFR_{K}}{\frac{\Delta V}{\Delta t}}\right)}\right]$ is $‐{\left(\frac{V_{0}}{V_{0}+\frac{\Delta V}{\Delta t}t}\right)}^{\left(1+\frac{GFR_{K}}{\frac{\Delta V}{\Delta t}}\right)}\cdot \frac{1}{\frac{\Delta V}{\Delta t}}\cdot \ln\left(\frac{V_{0}}{V_{0}+\frac{\Delta V}{\Delta t}t}\right)$. Next, the derivative of $\left(\frac{\mathrm{Gen}}{GFR_{K}+\frac{\Delta V}{\Delta t}}‐{\left[\mathrm{Cr}\right]}_{0}\right)$ is $\frac{‐Gen}{{\left(GFR_{K}+\frac{\Delta V}{\Delta t}\right)}^{2}}$. Putting it all together and using the product rule on Equation (2), we see that $\frac{\partial {\left[\mathrm{Cr}\right]}_{t}}{\partial GFR_{K}}$ is:

(3) $$\begin{array}{cc} \frac{\partial {\left[\mathrm{Cr}\right]}_{t}}{\partial GFR_{K}} & =\underset{a^{\prime }}{\underbrace{‐{\left(\frac{V_{0}}{V_{0}+\frac{\Delta V}{\Delta t}t}\right)}^{\left(1+\frac{GFR_{K}}{\frac{\Delta V}{\Delta t}}\right)}\cdot \frac{1}{\frac{\Delta V}{\Delta t}}\cdot \ln\left(\frac{V_{0}}{V_{0}+\frac{\Delta V}{\Delta t}t}\right)}}\\ & \;\times \underset{b}{\underbrace{\left(\frac{\mathrm{Gen}}{GFR_{K}+\frac{\Delta V}{\Delta t}}‐{\left[\mathrm{Cr}\right]}_{0}\right)}}\\ & \;+\underset{a}{\underbrace{\left[1‐{\left(\frac{V_{0}}{V_{0}+\frac{\Delta V}{\Delta t}t}\right)}^{\left(1+\frac{GFR_{K}}{\frac{\Delta V}{\Delta t}}\right)}\right]}}\cdot \underset{b^{\prime }}{\underbrace{\frac{‐Gen}{{\left(GFR_{K}+\frac{\Delta V}{\Delta t}\right)}^{2}}}}. \end{array}$$

### 2.2 Limits

The derivative in Equation (3) has three special cases that threaten a division by zero: (1) $\frac{\Delta V}{\Delta t}=0$, (2) $GFR_{K}=‐\frac{\Delta V}{\Delta t}$, and (3) $GFR_{K}=\frac{\Delta V}{\Delta t}=0$, which itself is a special case of (2). Each of these cases can be resolved by using limits. See Appendix for derivation details.

Case 1: $\frac{\Delta V}{\Delta t}=0.$

Of the three cases, this is the most important clinically, as a stable volume is common or at least is assumed when information on I's/O's is unavailable.

(4) $$\underset{\frac{\Delta V}{\Delta t}\to 0}{\text{lim}}\frac{\partial {\left[\mathrm{Cr}\right]}_{t}}{\partial GFR_{K}}=e^{\frac{‐GFR_{K}\cdot t}{V_{0}}}\cdot \frac{t}{V_{0}}\cdot \left(\frac{\mathrm{Gen}}{GFR_{K}}‐{\left[\mathrm{Cr}\right]}_{0}\right)‐\left(1‐e^{\frac{‐GFR_{K}\cdot t}{V_{0}}}\right)\cdot \frac{\mathrm{Gen}}{{\left(GFR_{K}\right)}^{2}}.$$

Case 2: $GFR_{K}=‐\frac{\Delta V}{\Delta t}$.

(5) $$\underset{GFR_{K}\to ‐\frac{\Delta V}{\Delta t}}{\text{lim}}\frac{\partial {\left[\mathrm{Cr}\right]}_{t}}{\partial GFR_{K}}=‐\frac{1}{\frac{\Delta V}{\Delta t}}\cdot \ln\left(\frac{V_{0}}{V_{0}+\frac{\Delta V}{\Delta t}t}\right)\cdot \left[\frac{\mathrm{Gen}}{2\cdot \frac{\Delta V}{\Delta t}}\cdot \ln\left(\frac{V_{0}}{V_{0}+\frac{\Delta V}{\Delta t}t}\right)‐{\left[\mathrm{Cr}\right]}_{0}\right].$$

Case 3: $GFR_{K}=\frac{\Delta V}{\Delta t}=0$.

(6) $$\underset{\left(GFR_{K},\frac{\Delta V}{\Delta t}\right)\to \left(0,0\right)}{\text{lim}}\frac{\partial {\left[\mathrm{Cr}\right]}_{t}}{\partial GFR_{K}}=‐\frac{t}{V_{0}}\cdot \left(\frac{Gen\cdot t}{2\cdot V_{0}}+{\left[\mathrm{Cr}\right]}_{0}\right).$$

## 3 RESULTS

### 3.1 Hypothesis plausibility

**FIGURE 2:**
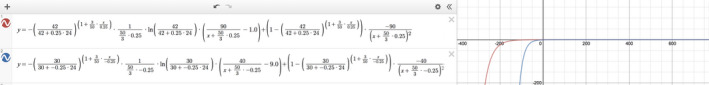
The partial derivative $\frac{\partial {\left[\mathrm{Cr}\right]}_{t}}{\partial GFR_{K}}$ in Equation (3) is graphed against $GFR_{K}$ as the independent variable. The red curve retains the miscellaneous clinical values as chosen in Figure 1. All of its y‐values $\left(\frac{\partial {\left[\mathrm{Cr}\right]}_{t}}{\partial GFR_{K}}\right)$ are negative, consistent with the hypothesis that changes in [creatinine] and kinetic GFR always move in opposite directions if all of the other variables are kept constant. The blue curve uses a more extreme but still allowable set of clinical values like ${\left[\mathrm{Cr}\right]}_{0}=9.0\;\text{mg}/\text{dL}$, $V_{0}=30\;L$, $\frac{\Delta V}{\Delta t}=‐0.25\;L/h$, Gen=40mg/dL·mL/min, while keeping t=24h. Even so, the blue curve's y‐values $\left(\frac{\partial {\left[\mathrm{Cr}\right]}_{t}}{\partial GFR_{K}}\right)$ are persistently negative.

To see if our intuition is on the right track that $\frac{\partial {\left[\mathrm{Cr}\right]}_{t}}{\partial GFR_{K}}$ is always negative, we graphed $\frac{\partial {\left[\mathrm{Cr}\right]}_{t}}{\partial GFR_{K}}$ $\left(y\right)$ versus $GFR_{K}$ $\left(x\right)$ (Figure 2). For the remaining variables, we kept them constant at clinically plausible values and tried various permutations, such as a low $V_{0}$, standard time, large $\frac{\Delta V}{\Delta t}$ (both ±), high ${\left[\mathrm{Cr}\right]}_{0}$, and small Gen, to see if we could turn $\frac{\partial {\left[\mathrm{Cr}\right]}_{t}}{\partial GFR_{K}}$ positive. No matter what combination was tried, $\frac{\partial {\left[\mathrm{Cr}\right]}_{t}}{\partial GFR_{K}}$ was always negative, i.e., below the x‐axis.

### 3.2 Rules for the proof

All of the variables have constraints on their values as imposed by clinical reality. The rules are:

A. Creatinine must be positive: ${\left[\mathrm{Cr}\right]}_{t}>0$ and ${\left[\mathrm{Cr}\right]}_{0}>0$.
B. Volume of distribution must be positive: $V_{0}>0$ and $V_{0}+\frac{\Delta V}{\Delta t}t>0$.
C. Kinetic GFR is traditionally non‐negative: $GFR_{K}\geq 0$.
D. The creatinine generation rate must be positive: Gen>0.
E. Volume change rate can be positive or negative, but it cannot be so negative in magnitude that a patient would lose all of the volume of distribution in an allotted time $\left(\frac{\Delta V}{\Delta t}>‐\frac{V_{0}}{t}\right).$ In theory, a positive volume change rate has no upper limit on magnitude $\left(\frac{\Delta V}{\Delta t}<+\infty \right)$.

### 3.3 Proof of the hypothesis

The derivative, rearranged to emphasize the leading negative sign, is hypothesized to always be negative for clinically realistic values, that is, the expression in the curly brace must be positive.

$$\begin{array}{cc} \frac{\partial {\left[\mathrm{Cr}\right]}_{t}}{\partial GFR_{K}} & =‐\left\{{\left(\frac{V_{0}}{V_{0}+\frac{\Delta V}{\Delta t}t}\right)}^{\left(1+\frac{GFR_{K}}{\frac{\Delta V}{\Delta t}}\right)}\cdot \frac{1}{\frac{\Delta V}{\Delta t}}\cdot \ln\left(\frac{V_{0}}{V_{0}+\frac{\Delta V}{\Delta t}t}\right)\cdot \left(\frac{\mathrm{Gen}}{GFR_{K}+\frac{\Delta V}{\Delta t}}‐{\left[\mathrm{Cr}\right]}_{0}\right)\right.\\ & \;\left.+\left[1‐{\left(\frac{V_{0}}{V_{0}+\frac{\Delta V}{\Delta t}t}\right)}^{\left(1+\frac{GFR_{K}}{\frac{\Delta V}{\Delta t}}\right)}\right]\cdot \frac{\mathrm{Gen}}{{\left(GFR_{K}+\frac{\Delta V}{\Delta t}\right)}^{2}}\right\}. \end{array}$$

The general strategy will be a proof by contradiction. We test whether the expression within the curly brace can be negative and prove that it cannot, in all six of the scenarios that are possible. Thus, the partial derivative $\frac{\partial {\left[\mathrm{Cr}\right]}_{t}}{\partial GFR_{K}}$ is always negative (except when it is zero at t=0, which is a trivial case). Due to its length, the complete proof is presented in the Appendix.

### 3.4 Where is the derivative bigger?

**FIGURE 3:**
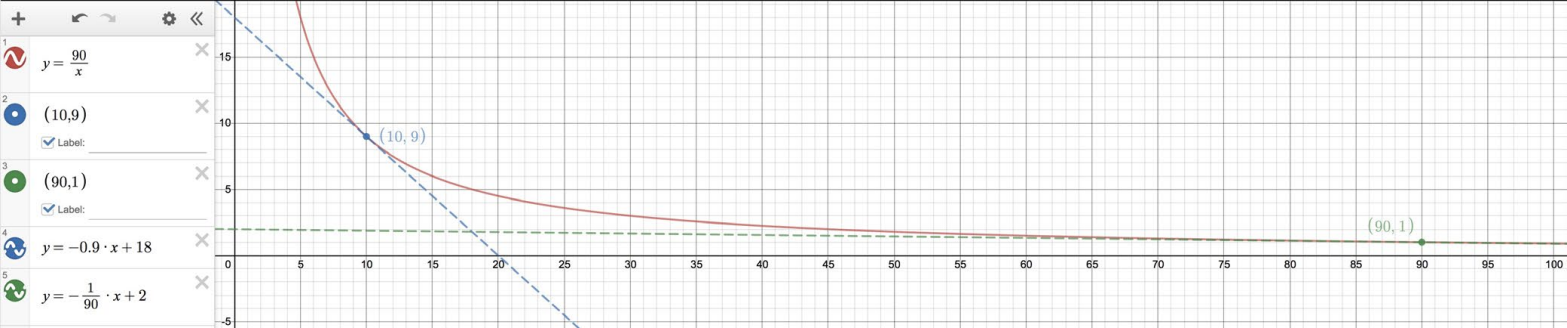
Analogous to $\frac{\partial {\left[\mathrm{Cr}\right]}_{t}}{\partial GFR_{K}}$, the $\frac{\Delta \left[\mathrm{Cr}\right]}{\Delta GFR}$ slopes in chronic kidney disease are also negative. In CKD, the [creatinine] (y‐axis) is simply the Gen $\left(\text{in this case}\;90\;\text{mg/dL}\cdot \text{mL/min}\right)$ divided by the GFR (x‐axis), a reciprocal function as shown by the red curve. At the low end of the [creatinine] scale, e.g., green dot $\left(\left[\mathrm{Cr}\right]=1\;\text{mg}/\text{dL}\right)$, the $\frac{\Delta \left[\mathrm{Cr}\right]}{\Delta GFR}$ slope is small in magnitude (−1/90, green dash line is tangent). At the high end of the [creatinine] scale, e.g., blue dot $\left(\left[\mathrm{Cr}\right]=9\;\text{mg}/\text{dL}\right)$, the $\frac{\Delta \left[\mathrm{Cr}\right]}{\Delta GFR}$ slope is much larger in magnitude (−0.9, blue dash line is tangent).

An observation familiar to nephrologists is that a small change at the low end of the [creatinine] scale represents a big change in GFR, whereas a big change at the high end of the [creatinine] scale represents a small change in GFR. A typical anecdote is that an increase in the [creatinine] from 1.0 to 2.0 mg/dL is akin to a 50% decrease in the GFR. But the same absolute increase in the [creatinine] from 9.0 to 10.0 mg/dL is only a 10% drop in the GFR. The statements assume that each [creatinine] was more or less in a steady state, which models a slowly progressive chronic kidney disease (CKD). In CKD, the creatinine excretion rate is nearly equal to but is slightly less than the creatinine production rate, which is why the serum [creatinine] slowly increases over time. Since the creatinine production rate is relatively constant if the patient's muscle mass remains stable, then the clearance expression $\frac{\overset{\begin{array}{c} \text{Creatinine}\\ \text{excretion rate} \end{array}}{\overbrace{U_{\mathrm{Cr}}\times V}}}{\underset{\begin{array}{c} \text{Plasma}\\ \text{creatinine} \end{array}}{\underbrace{P_{\mathrm{Cr}}}}}$ is more or less a constant divided by a [creatinine] that varies, i.e., a reciprocal function. On a graph of $\left[\mathrm{Cr}\right]$ versus GFR in CKD, at the low end of the $\left[\mathrm{Cr}\right]$ scale, a big loss of GFR (x‐axis) is needed to raise the $\left[\mathrm{Cr}\right]$ (y‐axis) even a tiny bit. That makes the $\frac{\overset{\text{tiny}}{\overbrace{\Delta \left[\mathrm{Cr}\right]}}}{\underset{\text{big}}{\underbrace{\Delta GFR}}}$ slope small, and negative, demonstrated in Figure 3. But at the high end of the $\left[\mathrm{Cr}\right]$ scale, just a tiny loss of GFR leverages a big rise in $\left[\mathrm{Cr}\right]$, making $\frac{\overset{\text{big}}{\overbrace{\Delta \left[\mathrm{Cr}\right]}}}{\underset{\text{tiny}}{\underbrace{\Delta GFR}}}$ large and, again, negative (Figure 3). Thus, a reciprocal function's very nature explains the anecdote observed in CKD.

**FIGURE 4:**
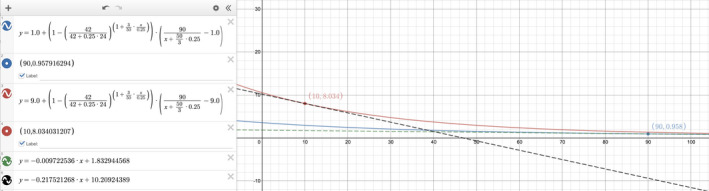
To see if acute kidney injury might follow a similar pattern as chronic kidney disease, we visualized the steepness of the $\frac{\partial {\left[\mathrm{Cr}\right]}_{t}}{\partial GFR_{K}}$ slope when the starting [creatinine], ${\left[\mathrm{Cr}\right]}_{0}$, is low versus high. When the kinetic GFR Equation (2) is graphed as $GFR_{K}$ on the x‐axis and ${\left[\mathrm{Cr}\right]}_{t}$ on the y‐axis, the blue curve shows the relationship when ${\left[\mathrm{Cr}\right]}_{0}=1.0$ and the red curve shows the relationship when ${\left[\mathrm{Cr}\right]}_{0}=9.0$. For a fair comparison, the miscellaneous clinical values are kept the same between the two curves. At the low end of the [creatinine] scale, the tangent at ${\left[\mathrm{Cr}\right]}_{t}=0.96\;\text{mg}/\text{dL}$ (blue dot) has a slope of ‐0.0097mg/dL per mL/min (green dash line). At the high end of the [creatinine] scale, the tangent at ${\left[\mathrm{Cr}\right]}_{t}=8.03\;\text{mg}/\text{dL}$ (red dot) has a slope of ‐0.2175mg/dL per mL/min (black dash line). Qualitatively, the pattern appears to be that a low [creatinine] gives a smaller $\frac{\partial {\left[\mathrm{Cr}\right]}_{t}}{\partial GFR_{K}}$ and that a high [creatinine] gives a larger $\frac{\partial {\left[\mathrm{Cr}\right]}_{t}}{\partial GFR_{K}}$, with all derivative signs being negative.

Do the slope lessons of CKD carry over into AKI? That is, does the AKI analog of $\frac{\Delta \left[\mathrm{Cr}\right]}{\Delta GFR}$, i.e., $\frac{\partial {\left[\mathrm{Cr}\right]}_{t}}{\partial GFR_{K}}$, follow the same pattern as before: low ${\left[\mathrm{Cr}\right]}_{0}$ ⇒ smaller $\frac{\partial {\left[\mathrm{Cr}\right]}_{t}}{\partial GFR_{K}}$ and high ${\left[\mathrm{Cr}\right]}_{0}$ ⇒ larger $\frac{\partial {\left[\mathrm{Cr}\right]}_{t}}{\partial GFR_{K}}$ (in magnitude)? Try a low initial $\left[\mathrm{Cr}\right]$ like 1.0 mg/dL. In steady state, that may correspond to a GFR of 90 mL/min if the creatinine generation rate were 90 mg/dL·mL/min. Let the kinetic GFR change from 90 mL/min and graph its effect on the ${\left[\mathrm{Cr}\right]}_{t}$, using Equation (2). For the other parameters of AKI, we kept t=24h and $V_{0}=42\;L$ and $\frac{\Delta V}{\Delta t}=0.25\;L/h$. As seen in Figure 4, the tangent to the curve at $GFR_{K}=90\;\text{mL}/\text{min}$ has a $\frac{\partial {\left[\mathrm{Cr}\right]}_{t}}{\partial GFR_{K}}$ slope that is shallow $\left(‐0.009722536\;\text{mg}/\text{dL per mL}/\text{min}\right)$. Now, try a high initial $\left[\mathrm{Cr}\right]$ like 9.0 mg/dL, which in steady state would correspond to a GFR of 10 mL/min. If we kept all other parameters the same, the tangent to this other curve at $GFR_{K}=10\;\text{mL}/\text{min}$ has a $\frac{\partial {\left[\mathrm{Cr}\right]}_{t}}{\partial GFR_{K}}$ slope that is steeper $\left(‐0.217521268\;\text{mg}/\text{dL per mL}/\text{min}\right)$ (Figure 4). This suggests that AKI recapitulates the behavior of CKD in terms of $\frac{\Delta \left[\mathrm{Cr}\right]}{\Delta GFR}$, albeit in a blunted way.

## 4 DISCUSSION

### 4.1 Clinical significance

The consistently negative value for $\frac{\partial {\left[\mathrm{Cr}\right]}_{t}}{\partial GFR_{K}}$ in the real world means that changes in kinetic GFR must always move in the opposite direction of changes in the serum [creatinine]. If the GFR goes up (even in the slightest), then more creatinine is excreted in that instant, and the [creatinine] must go down. Conversely, if the GFR goes down (even in the slightest), then less creatinine is excreted in that instant, and the [creatinine] must go up. Our proof of these assertions would affirm what most physicians intuitively sense about the relationship of GFR to the [creatinine]. Of course, this assumes that all of the other variables of creatinine kinetics like time and volume status remain constant, as is done when taking a partial derivative. All else being equal, a doctor can confidently know that the GFR can never change the [creatinine] in a parallel way. If at the 24‐h mark the [creatinine] were hypothetically greater than the actual—a positive $\Delta \left[\mathrm{Cr}\right]$—, then the GFR would have to be lower—a negative $\Delta GFR_{K}$.

### 4.2 Conundrum

One important lesson from applying the kinetic GFR to patient care was that an increasing [creatinine] does not always imply a decreasing GFR. Most of the time though, the two move in opposite directions, and this mental shortcut is used daily on the wards. However, there is a gray zone where some instances of a rising [creatinine] actually indicate an improving GFR. Let us say during an episode of AKI that a low GFR rises slightly. That would increase the creatinine excretion, but if that excretion remained less than the creatinine production, then the net *positive* balance would obligate the serum [creatinine] to rise still. It is not until the GFR increases by a sufficient amount to make the creatinine excretion equal to the creatinine production that the serum [creatinine] finally stabilizes. Thus, there is a transient period in renal recovery when the GFR is increasing and the [creatinine] is too. But did we not just prove that GFR cannot move in the same direction as [creatinine]? The conundrum is resolved by realizing that time is different in the two scenarios. When GFR and [creatinine] are both increasing, they are doing so relative to time. For example, the [creatinine] may have risen from 1.0 initially to 1.9 and then to 2.0 mg/dL $\left(↑\right)$, but the latter two values occurred at the 24‐ and 48‐h marks. Concordantly, the kinetic GFR may have been calculated to climb from 40 to 44 mL/min $\left(↑\right)$, but these are also at the 24‐ and 48‐h marks. In contrast, the derivative $\frac{\partial {\left[\mathrm{Cr}\right]}_{t}}{\partial GFR_{K}}$ assumes that time is a constant. It forces us to compare GFR and [creatinine] to themselves, but both at the 24‐h mark, for example. With that time constancy, if [creatinine] were to increase marginally, then GFR would have to *decrease* marginally. Furthermore, we could quantify that change by calculating $\frac{\partial {\left[\mathrm{Cr}\right]}_{t}}{\partial GFR_{K}}$'s value. Allowing the [creatinine], kinetic GFR, and time to all vary simultaneously is beyond the scope of this work.

### 4.3 What if *Gen* is changing?

Differential Equation (1) is set up to assume that the creatinine generation rate, Gen, is constant. That may be true for the most part, but Gen could be decreased during a critical illness such as sepsis (Doi et al., 2009; Prowle et al., 2014). More than an academic exercise, this finding has important clinical ramifications. If less creatinine is being produced, then a [creatinine] trajectory may not rise as quickly, and that will mute the apparent severity of the GFR loss during AKI, for example. Or, it could make the [creatinine] trajectory fall more quickly during a renal recovery, making the GFR gain seem more robust than it really is. Accounting for a changing Gen can potentially improve the accuracy of the kinetic GFR calculations. Unfortunately, it is not known how Gen evolves over time in most of our critically ill patients. We can measure it to be decreased, but was the evolution a sudden drop, a gradual and linear drop, a logistic model drop, etc.? Until patient data are gathered, it may be acceptable to treat a changing Gen as a sudden drop to a new value that remains stably low throughout the critical illness. If the acute drop is completed within 24 h, then the kinetic GFR calculations are easy to adapt. Just use the reduced Gen, whatever it is estimated (or, better yet, measured) to be, in the kinetic spreadsheet at the onset of and for the duration of the critical illness. That said, a Gen drop that is linear, like how volume change is modeled, can be incorporated into our differential Equation (1) to yield a closed‐form solution. However, its utility is limited to one or two rounds of calculation, since Gen cannot descend into negative values.

### 4.4 Conclusion

Doctors need to estimate the kidney function in the non‐steady state to care for their patients who develop acute renal failure. The GFR affects most facets of diagnosis and therapy, and the most cost‐effective way to estimate GFR at the bedside is to use the kinetic GFR equation (Endre et al., 2016; Khayat et al., 2019). Its math contains a lot of relationships that are waiting to be discovered. How the GFR influences the serum [creatinine] is important to investigate, and we can now prove what doctors intuitively think. If the GFR were to decrease further, then the [creatinine] would have to go up even more, and vice versa. In other words, their changes always move in opposite directions, as signified by the perpetual negative sign of $\frac{\partial {\left[\mathrm{Cr}\right]}_{t}}{\partial GFR_{K}}$. This does not contradict the paradoxical observations that an increase in serum [creatinine] can be compatible with a gain of kinetic GFR or that a decrease in [creatinine] can be compatible with a loss of kinetic GFR, because these changes are evolving over time. In contrast, time is fixed in the taking of the partial derivative.

### 4.5 Future work

Solving this derivative was just the beginning. We have since differentiated [creatinine] with respect to each of the other variables, and all of the other derivative pairs have been found via implicit differentiation. Likely, the most interesting derivative is $\frac{\partial {\left[\mathrm{Cr}\right]}_{t}}{\partial \frac{\Delta V}{\Delta t}}$. If $\frac{\Delta V}{\Delta t}$ is negative, the loss in volume concentrates the [creatinine] so that $\Delta \left[\mathrm{Cr}\right]$ is expected to be positive. Alternatively, if $\frac{\Delta V}{\Delta t}$ is positive, the gain in volume dilutes the [creatinine] so that $\Delta \left[\mathrm{Cr}\right]$ is felt to be negative. Do these thought experiments prove that yet another derivative is always negative? The answer is no, and the potentially positive partial derivative of ${\left[\mathrm{Cr}\right]}_{t}$ with respect to $\frac{\Delta V}{\Delta t}$ is (see Appendix):

$$\begin{array}{cc} \frac{\partial {\left[\mathrm{Cr}\right]}_{t}}{\partial \frac{\Delta V}{\Delta t}} & ={\left(\frac{V_{0}}{V_{0}+\frac{\Delta V}{\Delta t}t}\right)}^{\left(1+\frac{GFR_{K}}{\frac{\Delta V}{\Delta t}}\right)}\cdot \left[\frac{GFR_{K}}{{\left(\frac{\Delta V}{\Delta t}\right)}^{2}}\cdot \ln\left(\frac{V_{0}}{V_{0}+\frac{\Delta V}{\Delta t}t}\right)+\left(1+\frac{GFR_{K}}{\frac{\Delta V}{\Delta t}}\right)\cdot \frac{t}{V_{0}+\frac{\Delta V}{\Delta t}t}\right]\\ & \;\times \left(\frac{\mathrm{Gen}}{GFR_{K}+\frac{\Delta V}{\Delta t}}‐{\left[\mathrm{Cr}\right]}_{0}\right)+\left[1‐{\left(\frac{V_{0}}{V_{0}+\frac{\Delta V}{\Delta t}t}\right)}^{\left(1+\frac{GFR_{K}}{\frac{\Delta V}{\Delta t}}\right)}\right]\cdot \frac{‐Gen}{{\left(GFR_{K}+\frac{\Delta V}{\Delta t}\right)}^{2}}. \end{array}$$

## 1 LIMITS

From the kinetic GFR equation (Equation (2) in the main text), the derivative of the serum [creatinine] $\left({\left[\mathrm{Cr}\right]}_{t}\right)$ with respect to kinetic GFR $\left(GFR_{K}\right)$ is:

$$\begin{array}{cc} \frac{\partial {\left[\mathrm{Cr}\right]}_{t}}{\partial GFR_{K}} & =‐{\left(\frac{V_{0}}{V_{0}+\frac{\Delta V}{\Delta t}t}\right)}^{\left(1+\frac{GFR_{K}}{\frac{\Delta V}{\Delta t}}\right)}\cdot \frac{1}{\frac{\Delta V}{\Delta t}}\cdot \ln\left(\frac{V_{0}}{V_{0}+\frac{\Delta V}{\Delta t}t}\right)\cdot \left(\frac{\mathrm{Gen}}{GFR_{K}+\frac{\Delta V}{\Delta t}}‐{\left[\mathrm{Cr}\right]}_{0}\right)\\ & \;+\left[1‐{\left(\frac{V_{0}}{V_{0}+\frac{\Delta V}{\Delta t}t}\right)}^{\left(1+\frac{GFR_{K}}{\frac{\Delta V}{\Delta t}}\right)}\right]\cdot \frac{‐Gen}{{\left(GFR_{K}+\frac{\Delta V}{\Delta t}\right)}^{2}} \end{array}$$

The following three cases of a division by zero represent removable discontinuities that can be resolved by using limits.

Case 1: $\frac{\Delta V}{\Delta t}=0$.

We will solve the limit piecewise, proceeding through the various components of $\frac{\partial {\left[\mathrm{Cr}\right]}_{t}}{\partial GFR_{K}}$.

To begin with, the exponential can be resolved as:

$$\underset{\frac{\Delta V}{\Delta t}\to 0}{\text{lim}}{\left(\frac{V_{0}}{V_{0}+\frac{\Delta V}{\Delta t}t}\right)}^{\left(1+\frac{GFR_{K}}{\frac{\Delta V}{\Delta t}}\right)}=\exp\left[\left(1+\frac{GFR_{K}}{\frac{\Delta V}{\Delta t}}\right)\cdot \ln\left(\frac{V_{0}}{V_{0}+\frac{\Delta V}{\Delta t}t}\right)\right]=\exp\left[\left(1+\frac{GFR_{K}}{\frac{\Delta V}{\Delta t}}\right)\cdot \ln\left(1+\frac{‐\frac{\Delta V}{\Delta t}t}{V_{0}+\frac{\Delta V}{\Delta t}t}\right)\right]$$

The dominant term in the Taylor series for $\ln\left(1+\frac{‐\frac{\Delta V}{\Delta t}t}{V_{0}+\frac{\Delta V}{\Delta t}t}\right)$ is $\frac{‐\frac{\Delta V}{\Delta t}t}{V_{0}+\frac{\Delta V}{\Delta t}t}+\cdots$. Substitute this in:

$$\begin{array}{cc} \exp\left[\left(1+\frac{GFR_{K}}{\frac{\Delta V}{\Delta t}}\right)\cdot \left(‐\frac{\frac{\Delta V}{\Delta t}t}{V_{0}+\frac{\Delta V}{\Delta t}t}+\cdots \right)\right] & =\exp\left[\left(‐\frac{\frac{\Delta V}{\Delta t}t}{V_{0}+\frac{\Delta V}{\Delta t}t}‐\frac{GFR_{K}}{\frac{\Delta V}{\Delta t}}\cdot \frac{\frac{\Delta V}{\Delta t}t}{V_{0}+\frac{\Delta V}{\Delta t}t}+\cdots \right)\right]\\ & =\exp\left(‐\frac{\frac{\Delta V}{\Delta t}t}{V_{0}+\frac{\Delta V}{\Delta t}t}‐\frac{GFR_{K}\cdot t}{V_{0}+\frac{\Delta V}{\Delta t}t}+\cdots \right) \end{array}$$

Now, let $\frac{\Delta V}{\Delta t}$ go to zero, and the limit is seen to be:

$$\underset{\frac{\Delta V}{\Delta t}\to 0}{\text{lim}}{\left(\frac{V_{0}}{V_{0}+\frac{\Delta V}{\Delta t}t}\right)}^{\left(1+\frac{GFR_{K}}{\frac{\Delta V}{\Delta t}}\right)}=\exp\left(‐\frac{GFR_{K}\cdot t}{V_{0}}\right)=e^{\frac{‐GFR_{K}\cdot t}{V_{0}}}$$

Another part of the derivative affected by $\frac{\Delta V}{\Delta t}=0$ is $\frac{1}{\frac{\Delta V}{\Delta t}}\cdot \ln\left(\frac{V_{0}}{V_{0}+\frac{\Delta V}{\Delta t}t}\right)$. Again, using the dominant term in the Taylor series for $\ln\left(\frac{V_{0}}{V_{0}+\frac{\Delta V}{\Delta t}t}\right)$, the limit becomes:

$$\underset{\frac{\Delta V}{\Delta t}\to 0}{\text{lim}}\frac{1}{\frac{\Delta V}{\Delta t}}\cdot \ln\left(\frac{V_{0}}{V_{0}+\frac{\Delta V}{\Delta t}t}\right)=\underset{\frac{\Delta V}{\Delta t}\to 0}{\text{lim}}\frac{1}{\frac{\Delta V}{\Delta t}}\cdot \left(‐\frac{\frac{\Delta V}{\Delta t}t}{V_{0}+\frac{\Delta V}{\Delta t}t}+\cdots \right)=‐\frac{t}{V_{0}}$$

The rest of the derivative in the limit as $\frac{\Delta V}{\Delta t}$ approaches zero is straightforward:

(1) $$\underset{\frac{\Delta V}{\Delta t}\to 0}{\text{lim}}\frac{\partial {\left[\mathrm{Cr}\right]}_{t}}{\partial GFR_{K}}=e^{\frac{‐GFR_{K}\cdot t}{V_{0}}}\cdot \frac{t}{V_{0}}\cdot \left(\frac{\mathrm{Gen}}{GFR_{K}}‐{\left[\mathrm{Cr}\right]}_{0}\right)‐\left(1‐e^{\frac{‐GFR_{K}\cdot t}{V_{0}}}\right)\cdot \frac{\mathrm{Gen}}{{\left(GFR_{K}\right)}^{2}}.$$

Case 2: $GFR_{K}=‐\frac{\Delta V}{\Delta t}$.

Although it is unlikely that the $GFR_{K}$ will equal the negative of the volume change rate, the limit is interesting to solve. Letting $GFR_{K}$ approach $‐\frac{\Delta V}{\Delta t}$ is equivalent to stating that $GFR_{K}=‐\frac{\Delta V}{\Delta t}+h$, and letting *h* approach zero. This substitution transforms $\frac{\partial {\left[\mathrm{Cr}\right]}_{t}}{\partial GFR_{K}}$ into:

$$\frac{\partial {\left[\mathrm{Cr}\right]}_{t}}{\partial GFR_{K}}=‐{\left(\frac{V_{0}}{V_{0}+\frac{\Delta V}{\Delta t}t}\right)}^{\left(1+\frac{‐\frac{\Delta V}{\Delta t}+h}{\frac{\Delta V}{\Delta t}}\right)}\cdot \frac{1}{\frac{\Delta V}{\Delta t}}\cdot \ln\left(\frac{V_{0}}{V_{0}+\frac{\Delta V}{\Delta t}t}\right)\cdot \left(\frac{\mathrm{Gen}}{h}‐{\left[\mathrm{Cr}\right]}_{0}\right)+\left[1‐{\left(\frac{V_{0}}{V_{0}+\frac{\Delta V}{\Delta t}t}\right)}^{\left(1+\frac{‐\frac{\Delta V}{\Delta t}+h}{\frac{\Delta V}{\Delta t}}\right)}\right]\cdot \frac{‐Gen}{h^{2}}$$

$$\underset{h\to 0}{\text{lim}}\frac{\partial {\left[\mathrm{Cr}\right]}_{t}}{\partial GFR_{K}}=‐\underset{e^{0}\;\text{- like}}{\underbrace{{\left(\frac{V_{0}}{V_{0}+\frac{\Delta V}{\Delta t}t}\right)}^{\left(\frac{h}{\frac{\Delta V}{\Delta t}}\right)}}}\cdot \frac{1}{\frac{\Delta V}{\Delta t}}\cdot \ln\left(\frac{V_{0}}{V_{0}+\frac{\Delta V}{\Delta t}t}\right)\cdot \left(\frac{\mathrm{Gen}}{h}‐{\left[\mathrm{Cr}\right]}_{0}\right)+\left[1‐\underset{e^{0}\;\text{- like}}{\underbrace{{\left(\frac{V_{0}}{V_{0}+\frac{\Delta V}{\Delta t}t}\right)}^{\left(\frac{h}{\frac{\Delta V}{\Delta t}}\right)}}}\right]\cdot \frac{‐Gen}{h^{2}}$$

We will solve the limit piecewise. First, find the Taylor expansion of the $e^{0}$‐like expression:

$$\underset{h\to 0}{\text{lim}}{\left(\frac{V_{0}}{V_{0}+\frac{\Delta V}{\Delta t}t}\right)}^{\left(\frac{h}{\frac{\Delta V}{\Delta t}}\right)}=\exp\left[\frac{h}{\frac{\Delta V}{\Delta t}}\cdot \ln\left(\frac{V_{0}}{V_{0}+\frac{\Delta V}{\Delta t}t}\right)\right]=1+\frac{h}{\frac{\Delta V}{\Delta t}}\cdot \ln\left(\frac{V_{0}}{V_{0}+\frac{\Delta V}{\Delta t}t}\right)+\frac{\frac{h^{2}}{{\frac{\Delta V}{\Delta t}}^{2}}\cdot \ln^{2}\left(\frac{V_{0}}{V_{0}+\frac{\Delta V}{\Delta t}t}\right)}{2!}+\cdots$$

Substitute in the $e^{0}$‐like Taylor expansion, and the first half of the limit becomes:

$$‐\left[1+\frac{h}{\frac{\Delta V}{\Delta t}}\cdot \ln\left(\frac{V_{0}}{V_{0}+\frac{\Delta V}{\Delta t}t}\right)+\frac{h^{2}}{2\cdot {\frac{\Delta V}{\Delta t}}^{2}}\cdot \ln^{2}\left(\frac{V_{0}}{V_{0}+\frac{\Delta V}{\Delta t}t}\right)+\cdots \right]\cdot \frac{1}{\frac{\Delta V}{\Delta t}}\cdot \ln\left(\frac{V_{0}}{V_{0}+\frac{\Delta V}{\Delta t}t}\right)\cdot \left(\frac{\mathrm{Gen}}{h}‐{\left[\mathrm{Cr}\right]}_{0}\right)$$

$$‐\left[\frac{1}{\frac{\Delta V}{\Delta t}}\cdot \ln\left(\frac{V_{0}}{V_{0}+\frac{\Delta V}{\Delta t}t}\right)+\frac{h}{{\frac{\Delta V}{\Delta t}}^{2}}\cdot \ln^{2}\left(\frac{V_{0}}{V_{0}+\frac{\Delta V}{\Delta t}t}\right)+\frac{h^{2}}{2\cdot {\frac{\Delta V}{\Delta t}}^{3}}\cdot \ln^{3}\left(\frac{V_{0}}{V_{0}+\frac{\Delta V}{\Delta t}t}\right)+\cdots \right]\cdot \left(\frac{\mathrm{Gen}}{h}‐{\left[\mathrm{Cr}\right]}_{0}\right)$$

$$\left[\begin{array}{c} ‐\frac{\mathrm{Gen}}{h\cdot \frac{\Delta V}{\Delta t}}\cdot \ln\left(\frac{V_{0}}{V_{0}+\frac{\Delta V}{\Delta t}t}\right)‐\frac{\mathrm{Gen}}{{\frac{\Delta V}{\Delta t}}^{2}}\cdot \ln^{2}\left(\frac{V_{0}}{V_{0}+\frac{\Delta V}{\Delta t}t}\right)‐\frac{h\cdot Gen}{2\cdot {\frac{\Delta V}{\Delta t}}^{3}}\cdot \ln^{3}\left(\frac{V_{0}}{V_{0}+\frac{\Delta V}{\Delta t}t}\right)\\ +\frac{1}{\frac{\Delta V}{\Delta t}}\cdot \ln\left(\frac{V_{0}}{V_{0}+\frac{\Delta V}{\Delta t}t}\right)\cdot {\left[\mathrm{Cr}\right]}_{0}+\frac{h}{{\frac{\Delta V}{\Delta t}}^{2}}\cdot \ln^{2}\left(\frac{V_{0}}{V_{0}+\frac{\Delta V}{\Delta t}t}\right)\cdot {\left[\mathrm{Cr}\right]}_{0}+\frac{h^{2}}{2\cdot {\frac{\Delta V}{\Delta t}}^{3}}\cdot \ln^{3}\left(\frac{V_{0}}{V_{0}+\frac{\Delta V}{\Delta t}t}\right)\cdot {\left[\mathrm{Cr}\right]}_{0}+\cdots \end{array}\right]$$

Substitute in the $e^{0}$‐like Taylor expansion, and the second half of the limit becomes:

$$\left[1‐\left(1+\frac{h}{\frac{\Delta V}{\Delta t}}\cdot \ln\left(\frac{V_{0}}{V_{0}+\frac{\Delta V}{\Delta t}t}\right)+\frac{h^{2}}{2\cdot {\frac{\Delta V}{\Delta t}}^{2}}\cdot \ln^{2}\left(\frac{V_{0}}{V_{0}+\frac{\Delta V}{\Delta t}t}\right)+\frac{h^{3}}{6\cdot {\frac{\Delta V}{\Delta t}}^{3}}\cdot \ln^{3}\left(\frac{V_{0}}{V_{0}+\frac{\Delta V}{\Delta t}t}\right)+\cdots \right)\right]\cdot \frac{‐Gen}{h^{2}}$$

$$\left[‐\frac{h}{\frac{\Delta V}{\Delta t}}\cdot \ln\left(\frac{V_{0}}{V_{0}+\frac{\Delta V}{\Delta t}t}\right)‐\frac{h^{2}}{2\cdot {\frac{\Delta V}{\Delta t}}^{2}}\cdot \ln^{2}\left(\frac{V_{0}}{V_{0}+\frac{\Delta V}{\Delta t}t}\right)‐\frac{h^{3}}{6\cdot {\frac{\Delta V}{\Delta t}}^{3}}\cdot \ln^{3}\left(\frac{V_{0}}{V_{0}+\frac{\Delta V}{\Delta t}t}\right)+\cdots \right]\cdot \frac{‐Gen}{h^{2}}$$

$$\frac{\mathrm{Gen}}{h\cdot \frac{\Delta V}{\Delta t}}\cdot \ln\left(\frac{V_{0}}{V_{0}+\frac{\Delta V}{\Delta t}t}\right)+\frac{\mathrm{Gen}}{2\cdot {\frac{\Delta V}{\Delta t}}^{2}}\cdot \ln^{2}\left(\frac{V_{0}}{V_{0}+\frac{\Delta V}{\Delta t}t}\right)+\frac{h\cdot Gen}{6\cdot {\frac{\Delta V}{\Delta t}}^{3}}\cdot \ln^{3}\left(\frac{V_{0}}{V_{0}+\frac{\Delta V}{\Delta t}t}\right)+\cdots$$

Add the two halves of the limit together, and the problematic division by h→0 is subtracted out:

$$\left[\begin{array}{c} ‐\frac{\mathrm{Gen}}{2\cdot {\frac{\Delta V}{\Delta t}}^{2}}\cdot \ln^{2}\left(\frac{V_{0}}{V_{0}+\frac{\Delta V}{\Delta t}t}\right)‐\frac{h\cdot Gen}{3\cdot {\frac{\Delta V}{\Delta t}}^{3}}\cdot \ln^{3}\left(\frac{V_{0}}{V_{0}+\frac{\Delta V}{\Delta t}t}\right)\\ +\frac{1}{\frac{\Delta V}{\Delta t}}\cdot \ln\left(\frac{V_{0}}{V_{0}+\frac{\Delta V}{\Delta t}t}\right)\cdot {\left[\mathrm{Cr}\right]}_{0}+\frac{h}{{\frac{\Delta V}{\Delta t}}^{2}}\cdot \ln^{2}\left(\frac{V_{0}}{V_{0}+\frac{\Delta V}{\Delta t}t}\right)\cdot {\left[\mathrm{Cr}\right]}_{0}+\frac{h^{2}}{2\cdot {\frac{\Delta V}{\Delta t}}^{3}}\cdot \ln^{3}\left(\frac{V_{0}}{V_{0}+\frac{\Delta V}{\Delta t}t}\right)\cdot {\left[\mathrm{Cr}\right]}_{0}+\cdots \end{array}\right]$$

Now, let h go to zero, and the limit is seen to be:

$$‐\frac{\mathrm{Gen}}{2\cdot {\frac{\Delta V}{\Delta t}}^{2}}\cdot \ln^{2}\left(\frac{V_{0}}{V_{0}+\frac{\Delta V}{\Delta t}t}\right)+\frac{1}{\frac{\Delta V}{\Delta t}}\cdot \ln\left(\frac{V_{0}}{V_{0}+\frac{\Delta V}{\Delta t}t}\right)\cdot {\left[\mathrm{Cr}\right]}_{0}$$

(2) $$\underset{GFR_{K}\to ‐\frac{\Delta V}{\Delta t}}{\text{lim}}\frac{\partial {\left[\mathrm{Cr}\right]}_{t}}{\partial GFR_{K}}=‐\frac{1}{\frac{\Delta V}{\Delta t}}\cdot \ln\left(\frac{V_{0}}{V_{0}+\frac{\Delta V}{\Delta t}t}\right)\cdot \left[\frac{\mathrm{Gen}}{2\cdot \frac{\Delta V}{\Delta t}}\cdot \ln\left(\frac{V_{0}}{V_{0}+\frac{\Delta V}{\Delta t}t}\right)‐{\left[\mathrm{Cr}\right]}_{0}\right].$$

Case 3: $GFR_{K}=\frac{\Delta V}{\Delta t}=0$.

In the even more unlikely event that the kinetic GFR and the volume change rate are both zero, the dual limit approaching zero can be solved by at least two ways:

(1). Equation (1) was the limit of $\frac{\partial {\left[\mathrm{Cr}\right]}_{t}}{\partial GFR_{K}}$ as $\frac{\Delta V}{\Delta t}\to 0$. Now, take the limit as $GFR_{K}\to 0$:

$$\underset{\frac{\Delta V}{\Delta t}\to 0}{\text{lim}}\frac{\partial {\left[\mathrm{Cr}\right]}_{t}}{\partial GFR_{K}}=\underset{e^{0}\;\text{- like}}{\underbrace{e^{\frac{‐GFR_{K}\cdot t}{V_{0}}}}}\cdot \frac{t}{V_{0}}\cdot \left(\frac{\mathrm{Gen}}{GFR_{K}}‐{\left[\mathrm{Cr}\right]}_{0}\right)‐\left(1‐\underset{e^{0}\;\text{- like}}{\underbrace{e^{\frac{‐GFR_{K}\cdot t}{V_{0}}}}}\right)\cdot \frac{\mathrm{Gen}}{{\left(GFR_{K}\right)}^{2}}$$

The Taylor expansion of the $e^{0}$‐like term $e^{\frac{‐GFR_{K}\cdot t}{V_{0}}}$ is $1+\frac{‐GFR_{K}\cdot t}{V_{0}}+\frac{{\left(\frac{‐GFR_{K}\cdot t}{V_{0}}\right)}^{2}}{2!}+\cdots$ Substitute in:

$$\underset{\left(\frac{\Delta V}{\Delta t},GFR_{K}\right)\to \left(0,0\right)}{\text{lim}}\frac{\partial {\left[\mathrm{Cr}\right]}_{t}}{\partial GFR_{K}}=\left[1‐\frac{GFR_{K}\cdot t}{V_{0}}+\cdots \right]\cdot \frac{t}{V_{0}}\cdot \left(\frac{\mathrm{Gen}}{GFR_{K}}‐{\left[\mathrm{Cr}\right]}_{0}\right)\cdots$$

$$\cdots ‐\left(1‐\left[1‐\frac{GFR_{K}\cdot t}{V_{0}}+\frac{{\left(GFR_{K}\right)}^{2}\cdot t^{2}}{2\cdot {\left(V_{0}\right)}^{2}}+\cdots \right]\right)\cdot \frac{\mathrm{Gen}}{{\left(GFR_{K}\right)}^{2}}$$

$$\underset{\left(\frac{\Delta V}{\Delta t},GFR_{K}\right)\to \left(0,0\right)}{\text{lim}}\frac{\partial {\left[\mathrm{Cr}\right]}_{t}}{\partial GFR_{K}}=\left[\frac{t\cdot Gen}{V_{0}\cdot GFR_{K}}‐\frac{t}{V_{0}}\cdot {\left[\mathrm{Cr}\right]}_{0}‐\frac{t^{2}\cdot Gen}{{\left(V_{0}\right)}^{2}}+\frac{GFR_{K}\cdot t^{2}}{{\left(V_{0}\right)}^{2}}\cdot {\left[\mathrm{Cr}\right]}_{0}+\cdots \right]\cdots$$

$$\cdots ‐\left(\frac{GFR_{K}\cdot t}{V_{0}}‐\frac{{\left(GFR_{K}\right)}^{2}\cdot t^{2}}{2\cdot {\left(V_{0}\right)}^{2}}+\cdots \right)\cdot \frac{\mathrm{Gen}}{{\left(GFR_{K}\right)}^{2}}$$

The problematic division by $GFR_{K}\to 0$ is subtracted out:

$$\underset{\left(\frac{\Delta V}{\Delta t},GFR_{K}\right)\to \left(0,0\right)}{\text{lim}}\frac{\partial {\left[\mathrm{Cr}\right]}_{t}}{\partial GFR_{K}}=\frac{t\cdot Gen}{V_{0}\cdot GFR_{K}}‐\frac{t}{V_{0}}\cdot {\left[\mathrm{Cr}\right]}_{0}‐\frac{Gen\cdot t^{2}}{{\left(V_{0}\right)}^{2}}+\frac{GFR_{K}\cdot t^{2}}{{\left(V_{0}\right)}^{2}}\cdot {\left[\mathrm{Cr}\right]}_{0}‐\frac{t\cdot Gen}{V_{0}\cdot GFR_{K}}+\frac{Gen\cdot t^{2}}{2\cdot {\left(V_{0}\right)}^{2}}+\cdots$$

$$\underset{\left(\frac{\Delta V}{\Delta t},GFR_{K}\right)\to \left(0,0\right)}{\text{lim}}\frac{\partial {\left[\mathrm{Cr}\right]}_{t}}{\partial GFR_{K}}=‐\frac{t}{V_{0}}\cdot {\left[\mathrm{Cr}\right]}_{0}‐\frac{Gen\cdot t^{2}}{2\cdot {\left(V_{0}\right)}^{2}}+\frac{GFR_{K}\cdot t^{2}}{{\left(V_{0}\right)}^{2}}\cdot {\left[\mathrm{Cr}\right]}_{0}+\cdots$$

Now, let $GFR_{K}$ go to zero, and the limit is seen to be:

(3) $$\underset{\left(\frac{\Delta V}{\Delta t},GFR_{K}\right)\to \left(0,0\right)}{\text{lim}}\frac{\partial {\left[\mathrm{Cr}\right]}_{t}}{\partial GFR_{K}}=‐\frac{t}{V_{0}}\cdot {\left[\mathrm{Cr}\right]}_{0}‐\frac{Gen\cdot t^{2}}{2\cdot {\left(V_{0}\right)}^{2}}.$$

(2). The same limit as above would be found if Equation (2) had let $\frac{\Delta V}{\Delta t}$ approach zero:

$$\underset{GFR_{K}\to ‐\frac{\Delta V}{\Delta t}}{\text{lim}}\frac{\partial {\left[\mathrm{Cr}\right]}_{t}}{\partial GFR_{K}}=‐\frac{1}{\frac{\Delta V}{\Delta t}}\cdot \ln\left(\frac{V_{0}}{V_{0}+\frac{\Delta V}{\Delta t}t}\right)\cdot \left[\frac{\mathrm{Gen}}{2\cdot \frac{\Delta V}{\Delta t}}\cdot \ln\left(\frac{V_{0}}{V_{0}+\frac{\Delta V}{\Delta t}t}\right)‐{\left[\mathrm{Cr}\right]}_{0}\right]$$

Substitute in the Taylor expansion of $\ln\left(\frac{V_{0}}{V_{0}+\frac{\Delta V}{\Delta t}t}\right)$ as $\frac{\Delta V}{\Delta t}\to 0$:

$$\underset{\left(GFR_{K},\frac{\Delta V}{\Delta t}\right)\to \left(0,0\right)}{\text{lim}}\frac{\partial {\left[\mathrm{Cr}\right]}_{t}}{\partial GFR_{K}}=‐\frac{1}{\frac{\Delta V}{\Delta t}}\cdot \frac{‐\frac{\Delta V}{\Delta t}t}{V_{0}+\frac{\Delta V}{\Delta t}t}\cdot \left[\frac{\mathrm{Gen}}{2\cdot \frac{\Delta V}{\Delta t}}\cdot \frac{‐\frac{\Delta V}{\Delta t}t}{V_{0}+\frac{\Delta V}{\Delta t}t}‐{\left[\mathrm{Cr}\right]}_{0}\right]$$

$$\underset{\left(GFR_{K},\frac{\Delta V}{\Delta t}\right)\to \left(0,0\right)}{\text{lim}}\frac{\partial {\left[\mathrm{Cr}\right]}_{t}}{\partial GFR_{K}}=\frac{t}{V_{0}+\frac{\Delta V}{\Delta t}t}\cdot \left[‐\frac{Gen\cdot t}{2\cdot \left(V_{0}+\frac{\Delta V}{\Delta t}t\right)}‐{\left[\mathrm{Cr}\right]}_{0}\right]$$

Now, let $\frac{\Delta V}{\Delta t}$ go to zero, and the limit is seen to be:

(4) $$\underset{\left(GFR_{K},\frac{\Delta V}{\Delta t}\right)\to \left(0,0\right)}{\text{lim}}\frac{\partial {\left[\mathrm{Cr}\right]}_{t}}{\partial GFR_{K}}=‐\frac{t}{V_{0}}\cdot \left(\frac{Gen\cdot t}{2\cdot V_{0}}+{\left[\mathrm{Cr}\right]}_{0}\right).$$

The dual limits in Equations (3) and (4) are mathematically equivalent, which also validates the precursor limits in Equation (1): stable volume $\left(\frac{\Delta V}{\Delta t}=0\right)$ and Equation (2): $GFR_{K}=‐\frac{\Delta V}{\Delta t}$.

A fourth case of division by zero, $V_{0}+\frac{\Delta V}{\Delta t}t=0$, has a limit that is undefined, which is just as well since patients cannot have a zero volume.

## 2 Proof that $\frac{\partial {\left[\mathrm{Cr}\right]}_{t}}{\partial {\mathrm{GFR}}_{K}}$ is always negative

The derivative, rearranged to emphasize the leading negative sign, is hypothesized to always be negative for clinically realistic values, i.e., the expression in the curly brace must be positive.

$$\frac{\partial {\left[\mathrm{Cr}\right]}_{t}}{\partial GFR_{K}}=‐\left\{{\left(\frac{V_{0}}{V_{0}+\frac{\Delta V}{\Delta t}t}\right)}^{\left(1+\frac{GFR_{K}}{\frac{\Delta V}{\Delta t}}\right)}\cdot \frac{1}{\frac{\Delta V}{\Delta t}}\cdot \ln\left(\frac{V_{0}}{V_{0}+\frac{\Delta V}{\Delta t}t}\right)\cdot \left(\frac{\mathrm{Gen}}{GFR_{K}+\frac{\Delta V}{\Delta t}}‐{\left[\mathrm{Cr}\right]}_{0}\right)+\left[1‐{\left(\frac{V_{0}}{V_{0}+\frac{\Delta V}{\Delta t}t}\right)}^{\left(1+\frac{GFR_{K}}{\frac{\Delta V}{\Delta t}}\right)}\right]\cdot \frac{\mathrm{Gen}}{{\left(GFR_{K}+\frac{\Delta V}{\Delta t}\right)}^{2}}\right\}$$

The general strategy will be a proof by contradiction. We test whether the expression within the curly brace can be negative and prove that it cannot, in all six of the scenarios that are possible.

The curly brace's sum breaks up into two groups:

## 3 **GROUP 1**:

(5)
$$\underset{\text{always positive}}{\underbrace{{\left(\frac{V_{0}}{V_{0}+\frac{\Delta V}{\Delta t}t}\right)}^{\left(1+\frac{GFR_{K}}{\frac{\Delta V}{\Delta t}}\right)}}}\cdot \underset{\text{always negative}}{\underbrace{\frac{1}{\frac{\Delta V}{\Delta t}}\cdot \ln\left(\frac{V_{0}}{V_{0}+\frac{\Delta V}{\Delta t}t}\right)}}\cdot \underset{\text{can be}\;\pm \text{; determines sign}}{\underbrace{\left(\frac{\mathrm{Gen}}{GFR_{K}+\frac{\Delta V}{\Delta t}}‐{\left[\mathrm{Cr}\right]}_{0}\right)}}.$$

The ratio of initial‐to‐final volume $\frac{V_{0}}{V_{0}+\frac{\Delta V}{\Delta t}t}$ must be positive, and a positive number raised to any real exponent is also positive.

In $\frac{1}{\frac{\Delta V}{\Delta t}}\cdot \ln\left(\frac{V_{0}}{V_{0}+\frac{\Delta V}{\Delta t}t}\right)$, a negative $\frac{\Delta V}{\Delta t}$ makes the fraction $\frac{V_{0}}{V_{0}+\frac{\Delta V}{\Delta t}t}>1$, which makes its logarithm positive. Here, the product $\frac{1}{\frac{\Delta V}{\Delta t}}\cdot \ln\left(\frac{V_{0}}{V_{0}+\frac{\Delta V}{\Delta t}t}\right)$ is negative. On the other hand, a positive $\frac{\Delta V}{\Delta t}$ makes the fraction $\frac{V_{0}}{V_{0}+\frac{\Delta V}{\Delta t}t}<1$, which makes its logarithm negative. Either way, the product $\frac{1}{\frac{\Delta V}{\Delta t}}\cdot \ln\left(\frac{V_{0}}{V_{0}+\frac{\Delta V}{\Delta t}t}\right)$ is negative. (A zero $\frac{\Delta V}{\Delta t}$ is handled separately.)

The $\frac{\mathrm{Gen}}{GFR_{K}+\frac{\Delta V}{\Delta t}}‐{\left[\mathrm{Cr}\right]}_{0}$ represents the spread between the initial [creatinine] and the eventual steady‐state [creatinine]. If the [creatinine] is rising, the spread is positive. If the [creatinine] is falling, the spread is negative. Since $\frac{\mathrm{Gen}}{GFR_{K}+\frac{\Delta V}{\Delta t}}‐{\left[\mathrm{Cr}\right]}_{0}$ can take on either sign, it determines the overall sign of the three‐term product in Group 1.

## 4 **GROUP 2**:

(6)
$$\underset{\text{always positive}}{\underbrace{\frac{\mathrm{Gen}}{{\left(GFR_{K}+\frac{\Delta V}{\Delta t}\right)}^{2}}}}\cdot \underset{\text{can be}\;\pm \text{; determines sign}}{\underbrace{\left[1‐{\left(\frac{V_{0}}{V_{0}+\frac{\Delta V}{\Delta t}t}\right)}^{\left(1+\frac{GFR_{K}}{\frac{\Delta V}{\Delta t}}\right)}\right]}}.$$

In $\frac{\mathrm{Gen}}{{\left(GFR_{K}+\frac{\Delta V}{\Delta t}\right)}^{2}}$, Gen is positive, and the square ${\left(GFR_{K}+\frac{\Delta V}{\Delta t}\right)}^{2}$ is positive in the real numbers.

In $1‐{\left(\frac{V_{0}}{V_{0}+\frac{\Delta V}{\Delta t}t}\right)}^{\left(1+\frac{GFR_{K}}{\frac{\Delta V}{\Delta t}}\right)}$, a positive $\frac{\Delta V}{\Delta t}$ makes $0<\frac{V_{0}}{V_{0}+\frac{\Delta V}{\Delta t}t}<1$ and the exponent $1+\frac{GFR_{K}}{\frac{\Delta V}{\Delta t}}>0$. A base between zero and one raised to a positive exponent has a range between zero and one as well. Subsequently, $1‐{\left(\frac{V_{0}}{V_{0}+\frac{\Delta V}{\Delta t}t}\right)}^{\left(1+\frac{GFR_{K}}{\frac{\Delta V}{\Delta t}}\right)}$ is positive.

However, a negative $\frac{\Delta V}{\Delta t}$ makes $\frac{V_{0}}{V_{0}+\frac{\Delta V}{\Delta t}t}>1$, and raising this to any positive or negative exponent $1+\frac{GFR_{K}}{\frac{\Delta V}{\Delta t}}$ makes it positive and potentially greater than one. Subsequently, $1‐{\left(\frac{V_{0}}{V_{0}+\frac{\Delta V}{\Delta t}t}\right)}^{\left(1+\frac{GFR_{K}}{\frac{\Delta V}{\Delta t}}\right)}$ can be either positive or negative, thus determining the overall sign of Group 2.

Broadly, there are 3 ways to falsify the hypothesis by having a curly brace value that is negative. Referring to expression (5)—Group 1—and expression (6)—Group 2, the ways are:

1. Positive or zero $\frac{\mathrm{Gen}}{GFR_{K}+\frac{\Delta V}{\Delta t}}‐{\left[\mathrm{Cr}\right]}_{0}$ in Group 1 and Negative $1‐{\left(\frac{V_{0}}{V_{0}+\frac{\Delta V}{\Delta t}t}\right)}^{\left(1+\frac{GFR_{K}}{\frac{\Delta V}{\Delta t}}\right)}$ in Group 2.

2. Positive $\frac{\mathrm{Gen}}{GFR_{K}+\frac{\Delta V}{\Delta t}}‐{\left[\mathrm{Cr}\right]}_{0}$ and Positive $1‐{\left(\frac{V_{0}}{V_{0}+\frac{\Delta V}{\Delta t}t}\right)}^{\left(1+\frac{GFR_{K}}{\frac{\Delta V}{\Delta t}}\right)}$ and $\underset{\text{3rd condition}}{\underbrace{\left|\text{Group}1\right|>\left|\text{Group}2\right|}}$.

3. Negative $\frac{\mathrm{Gen}}{GFR_{K}+\frac{\Delta V}{\Delta t}}‐{\left[\mathrm{Cr}\right]}_{0}$ and Negative $1‐{\left(\frac{V_{0}}{V_{0}+\frac{\Delta V}{\Delta t}t}\right)}^{\left(1+\frac{GFR_{K}}{\frac{\Delta V}{\Delta t}}\right)}$ and $\underset{\text{3rd condition}}{\underbrace{\left|\text{Group}1\right|<\left|\text{Group}2\right|}}$.

Test Each Falsifying Way:

1. If $\frac{\mathrm{Gen}}{GFR_{K}+\frac{\Delta V}{\Delta t}}‐{\left[\mathrm{Cr}\right]}_{0}\geq 0$, then $‐GFR_{K}<\frac{\Delta V}{\Delta t}\leq ‐GFR_{K}+\frac{\mathrm{Gen}}{{\left[\mathrm{Cr}\right]}_{0}}$. (Note: $\frac{\Delta V}{\Delta t}=0$ is covered separately.)

Substitute these $\frac{\Delta V}{\Delta t}$’s into the exponent $1+\frac{GFR_{K}}{\frac{\Delta V}{\Delta t}}$ to get the two bounds: zero on one end and $\frac{\frac{\mathrm{Gen}}{{\left[\mathrm{Cr}\right]}_{0}}}{\frac{\Delta V}{\Delta t}}$, after simplifying and back‐substituting, on the other end.

Analyze the more interesting bound on the exponent: $1‐{\left(\frac{V_{0}}{V_{0}+\frac{\Delta V}{\Delta t}t}\right)}^{\left(\frac{\frac{\mathrm{Gen}}{{\left[\mathrm{Cr}\right]}_{0}}}{\frac{\Delta V}{\Delta t}}\right)}$:

i. If $\frac{\Delta V}{\Delta t}$ is negative, then $1‐{\underset{>1}{\underbrace{\left(\frac{V_{0}}{V_{0}+\frac{\Delta V}{\Delta t}t}\right)}}}^{\underset{\text{Negative}}{\underbrace{\left(\frac{\frac{\mathrm{Gen}}{{\left[\mathrm{Cr}\right]}_{0}}}{\frac{\Delta V}{\Delta t}}\right)}}}$ and the whole expression is positive, specifically that $0<1‐{\left(\frac{V_{0}}{V_{0}+\frac{\Delta V}{\Delta t}t}\right)}^{\left(\frac{\frac{\mathrm{Gen}}{{\left[\mathrm{Cr}\right]}_{0}}}{\frac{\Delta V}{\Delta t}}\right)}<1$.

ii. If $\frac{\Delta V}{\Delta t}$ is positive, then $1‐{\underset{<1}{\underbrace{\left(\frac{V_{0}}{V_{0}+\frac{\Delta V}{\Delta t}t}\right)}}}^{\underset{\text{Positive}}{\underbrace{\left(\frac{\frac{\mathrm{Gen}}{{\left[\mathrm{Cr}\right]}_{0}}}{\frac{\Delta V}{\Delta t}}\right)}}}$ and the whole expression is positive, specifically that $0<1‐{\left(\frac{V_{0}}{V_{0}+\frac{\Delta V}{\Delta t}t}\right)}^{\left(\frac{\frac{\mathrm{Gen}}{{\left[\mathrm{Cr}\right]}_{0}}}{\frac{\Delta V}{\Delta t}}\right)}<1$ again.

In both cases, the $1‐{\left(\frac{V_{0}}{V_{0}+\frac{\Delta V}{\Delta t}t}\right)}^{\left(1+\frac{GFR_{K}}{\frac{\Delta V}{\Delta t}}\right)}$ was positive, but it needed to be negative. Therefore, Way #1 does not falsify the hypothesis.

2. As learned in 1, if $\frac{\mathrm{Gen}}{GFR_{K}+\frac{\Delta V}{\Delta t}}‐{\left[\mathrm{Cr}\right]}_{0}>0$, then $1‐{\left(\frac{V_{0}}{V_{0}+\frac{\Delta V}{\Delta t}t}\right)}^{\left(1+\frac{GFR_{K}}{\frac{\Delta V}{\Delta t}}\right)}$ has to be positive. Together, they satisfy two out of the three conditions in 2. The 3rd condition, then, is key.

Requiring $\left|\text{Group 1}\right|>\left|\text{Group 2}\right|$ is equivalent to asking if

$\begin{array}{cc} & \underset{\text{Group 1 negative}}{\underbrace{{\left(\frac{V_{0}}{V_{0}+\frac{\Delta V}{\Delta t}t}\right)}^{\left(1+\frac{GFR_{K}}{\frac{\Delta V}{\Delta t}}\right)}\cdot \frac{1}{\frac{\Delta V}{\Delta t}}\cdot \ln\left(\frac{V_{0}}{V_{0}+\frac{\Delta V}{\Delta t}t}\right)\cdot \left(\frac{\mathrm{Gen}}{GFR_{K}+\frac{\Delta V}{\Delta t}}‐{\left[\mathrm{Cr}\right]}_{0}\right)}}\\ & \;+\underset{\text{Group 2 positive}}{\underbrace{\frac{\mathrm{Gen}}{{\left(GFR_{K}+\frac{\Delta V}{\Delta t}\right)}^{2}}\cdot \left[1‐{\left(\frac{V_{0}}{V_{0}+\frac{\Delta V}{\Delta t}t}\right)}^{\left(1+\frac{GFR_{K}}{\frac{\Delta V}{\Delta t}}\right)}\right]}}<0 \end{array}$

Given that $\frac{\mathrm{Gen}}{GFR_{K}+\frac{\Delta V}{\Delta t}}>{\left[\mathrm{Cr}\right]}_{0}$, let $\frac{\mathrm{Gen}}{GFR_{K}+\frac{\Delta V}{\Delta t}}=a{\left[\mathrm{Cr}\right]}_{0}$. Then a>1.

Also, since $\frac{\mathrm{Gen}}{GFR_{K}+\frac{\Delta V}{\Delta t}}$ is greater than a positive value of ${\left[\mathrm{Cr}\right]}_{0}$, then $GFR_{K}+\frac{\Delta V}{\Delta t}>0$.

In Group 2, $\frac{\mathrm{Gen}}{{\left(GFR_{K}+\frac{\Delta V}{\Delta t}\right)}^{2}}=\underset{=a{\left[\mathrm{Cr}\right]}_{0}}{\underbrace{\frac{\mathrm{Gen}}{GFR_{K}+\frac{\Delta V}{\Delta t}}}}\cdot \frac{1}{GFR_{K}+\frac{\Delta V}{\Delta t}}=\frac{a{\left[\mathrm{Cr}\right]}_{0}}{GFR_{K}+\frac{\Delta V}{\Delta t}}$. Substitute these in:

$${\left(\frac{V_{0}}{V_{0}+\frac{\Delta V}{\Delta t}t}\right)}^{\left(1+\frac{GFR_{K}}{\frac{\Delta V}{\Delta t}}\right)}\cdot \frac{1}{\frac{\Delta V}{\Delta t}}\cdot \ln\left(\frac{V_{0}}{V_{0}+\frac{\Delta V}{\Delta t}t}\right)\cdot \left(a{\left[\mathrm{Cr}\right]}_{0}‐{\left[\mathrm{Cr}\right]}_{0}\right)+\frac{a{\left[\mathrm{Cr}\right]}_{0}}{GFR_{K}+\frac{\Delta V}{\Delta t}}\cdot \left[1‐{\left(\frac{V_{0}}{V_{0}+\frac{\Delta V}{\Delta t}t}\right)}^{\left(1+\frac{GFR_{K}}{\frac{\Delta V}{\Delta t}}\right)}\right]<0$$

$${\left(\frac{V_{0}}{V_{0}+\frac{\Delta V}{\Delta t}t}\right)}^{\left(1+\frac{GFR_{K}}{\frac{\Delta V}{\Delta t}}\right)}\cdot \frac{1}{\frac{\Delta V}{\Delta t}}\cdot \ln\left(\frac{V_{0}}{V_{0}+\frac{\Delta V}{\Delta t}t}\right)\cdot \left(a‐1\right)+\underset{\text{Positive}}{\underbrace{\frac{a}{GFR_{K}+\frac{\Delta V}{\Delta t}}}}\cdot \left[1‐{\left(\frac{V_{0}}{V_{0}+\frac{\Delta V}{\Delta t}t}\right)}^{\left(1+\frac{GFR_{K}}{\frac{\Delta V}{\Delta t}}\right)}\right]<0$$

$${\left(\frac{V_{0}}{V_{0}+\frac{\Delta V}{\Delta t}t}\right)}^{\left(1+\frac{GFR_{K}}{\frac{\Delta V}{\Delta t}}\right)}\cdot \frac{GFR_{K}+\frac{\Delta V}{\Delta t}}{\frac{\Delta V}{\Delta t}}\cdot \ln\left(\frac{V_{0}}{V_{0}+\frac{\Delta V}{\Delta t}t}\right)\cdot \frac{a‐1}{a}+1‐{\left(\frac{V_{0}}{V_{0}+\frac{\Delta V}{\Delta t}t}\right)}^{\left(1+\frac{GFR_{K}}{\frac{\Delta V}{\Delta t}}\right)}<0$$

$$e^{\left(1+\frac{GFR_{K}}{\frac{\Delta V}{\Delta t}}\right)\cdot \ln\left(\frac{V_{0}}{V_{0}+\frac{\Delta V}{\Delta t}t}\right)}\cdot \left(1+\frac{GFR_{K}}{\frac{\Delta V}{\Delta t}}\right)\cdot \ln\left(\frac{V_{0}}{V_{0}+\frac{\Delta V}{\Delta t}t}\right)\cdot \frac{a‐1}{a}+1‐e^{\left(1+\frac{GFR_{K}}{\frac{\Delta V}{\Delta t}}\right)\cdot \ln\left(\frac{V_{0}}{V_{0}+\frac{\Delta V}{\Delta t}t}\right)}<0$$

Let $\left(1+\frac{GFR_{K}}{\frac{\Delta V}{\Delta t}}\right)\cdot \ln\left(\frac{V_{0}}{V_{0}+\frac{\Delta V}{\Delta t}t}\right)=‐x$. Because $GFR_{K}+\frac{\Delta V}{\Delta t}>0$, we restrict $\frac{\Delta V}{\Delta t}>‐GFR_{K}$. If $\frac{\Delta V}{\Delta t}$ is negative, then $\frac{GFR_{K}}{\frac{\Delta V}{\Delta t}}<‐1$ and $1+\frac{GFR_{K}}{\frac{\Delta V}{\Delta t}}$ is negative. If $\frac{\Delta V}{\Delta t}$ is positive, then $1+\frac{GFR_{K}}{\frac{\Delta V}{\Delta t}}$ is positive. Whichever sign $1+\frac{GFR_{K}}{\frac{\Delta V}{\Delta t}}$ has, the $\ln\left(\frac{V_{0}}{V_{0}+\frac{\Delta V}{\Delta t}t}\right)$ must take on the opposite sign, making $\underset{\text{Neg/Pos}}{\underbrace{\left(1+\frac{GFR_{K}}{\frac{\Delta V}{\Delta t}}\right)}}\cdot \underset{\text{Pos/Neg}}{\underbrace{\ln\left(\frac{V_{0}}{V_{0}+\frac{\Delta V}{\Delta t}t}\right)}}$ negative either way, so x has to be positive $\left(x>0\right)$.

(Note: $\frac{\Delta V}{\Delta t}=‐GFR_{K}$ and $\frac{\Delta V}{\Delta t}=GFR_{K}=0$ are covered separately.)

Substitute in: $e^{‐x}\cdot ‐x\cdot \frac{a‐1}{a}+1‐e^{‐x}<0$.

Multiply both sides by $e^{x}$: $‐x\cdot \frac{a‐1}{a}+e^{x}‐1<0$.

Let $f\left(x\right)=e^{x}‐\frac{a‐1}{a}\cdot x‐1$. Is this function always negative when x>0

Differentiate the function: $f^{\prime }\left(x\right)=e^{x}‐\frac{a‐1}{a}$.

Because x>0, then $e^{x}>1$. In contrast, because a>1, then $0<\frac{a‐1}{a}<1$.

The subtraction yields an $f^{\prime }\left(x\right)$ that is positive, meaning that $f\left(x\right)$ is increasing on x>0.

As x approaches zero from the right, $f\left(x\right)$ must have a minimum of $\underset{x\to 0^{+}}{\text{lim}}e^{x}‐\frac{a‐1}{a}\cdot x‐1=0$.

Thus, $f\left(x\right)$ is always positive, but that contradicts $f\left(x\right)=e^{x}‐\frac{a‐1}{a}\cdot x‐1$ needing to be negative, so Way #2 does not falsify the hypothesis.

3. If $\frac{\mathrm{Gen}}{GFR_{K}+\frac{\Delta V}{\Delta t}}‐{\left[\mathrm{Cr}\right]}_{0}<0$, then $\frac{\Delta V}{\Delta t}<\underset{‐\;\text{or}\;0}{\underbrace{‐GFR_{K}}}$ OR $‐GFR_{K}+\underset{+}{\underbrace{\frac{\mathrm{Gen}}{{\left[\mathrm{Cr}\right]}_{0}}}}<\frac{\Delta V}{\Delta t}$.

i. If $\frac{\Delta V}{\Delta t}<‐GFR_{K}$, then $1‐{\underset{>1}{\underbrace{\left(\frac{V_{0}}{V_{0}+\frac{\Delta V}{\Delta t}t}\right)}}}^{\underset{\text{Positive}}{\underbrace{\left(1+\frac{GFR_{K}}{\frac{\Delta V}{\Delta t}}\right)}}}$ is negative, as required in 3. OR:

ii. If $‐GFR_{K}+\frac{\mathrm{Gen}}{{\left[\mathrm{Cr}\right]}_{0}}<\frac{\Delta V}{\Delta t}<0$, then $1‐{\underset{>1}{\underbrace{\left(\frac{V_{0}}{V_{0}+\frac{\Delta V}{\Delta t}t}\right)}}}^{\underset{\text{Negative}}{\underbrace{\left(1+\frac{GFR_{K}}{\frac{\Delta V}{\Delta t}}\right)}}}$ is positive.

(Note: $\frac{\Delta V}{\Delta t}=0$ is covered separately.)

iii. If $0\leq ‐GFR_{K}+\frac{\mathrm{Gen}}{{\left[\mathrm{Cr}\right]}_{0}}<\frac{\Delta V}{\Delta t}$, then $1‐{\underset{<1}{\underbrace{\left(\frac{V_{0}}{V_{0}+\frac{\Delta V}{\Delta t}t}\right)}}}^{\underset{\text{Positive}}{\underbrace{\left(1+\frac{GFR_{K}}{\frac{\Delta V}{\Delta t}}\right)}}}$ is positive.

The latter two cases violate the 2nd condition that $1‐{\left(\frac{V_{0}}{V_{0}+\frac{\Delta V}{\Delta t}t}\right)}^{\left(1+\frac{GFR_{K}}{\frac{\Delta V}{\Delta t}}\right)}$ be negative, so only the first case of $\frac{\Delta V}{\Delta t}<‐GFR_{K}$ needs to be considered.

Once again, the 3rd condition is key, but this time $\left|\text{Group 1}\right|<\left|\text{Group 2}\right|$.

Requiring $\left|\text{Group 1}\right|<\left|\text{Group 2}\right|$ is equivalent to asking if

$\begin{array}{cc} & \underset{\text{Group 1 positive}}{\underbrace{{\left(\frac{V_{0}}{V_{0}+\frac{\Delta V}{\Delta t}t}\right)}^{\left(1+\frac{GFR_{K}}{\frac{\Delta V}{\Delta t}}\right)}\cdot \frac{1}{\frac{\Delta V}{\Delta t}}\cdot \ln\left(\frac{V_{0}}{V_{0}+\frac{\Delta V}{\Delta t}t}\right)\cdot \left(\frac{\mathrm{Gen}}{GFR_{K}+\frac{\Delta V}{\Delta t}}‐{\left[\mathrm{Cr}\right]}_{0}\right)}}\\ & \;+\underset{\text{Group 2 negative}}{\underbrace{\frac{\mathrm{Gen}}{{\left(GFR_{K}+\frac{\Delta V}{\Delta t}\right)}^{2}}\cdot \left[1‐{\left(\frac{V_{0}}{V_{0}+\frac{\Delta V}{\Delta t}t}\right)}^{\left(1+\frac{GFR_{K}}{\frac{\Delta V}{\Delta t}}\right)}\right]}}<0 \end{array}$

So if $\frac{\mathrm{Gen}}{GFR_{K}+\frac{\Delta V}{\Delta t}}<{\left[\mathrm{Cr}\right]}_{0}$, let $\frac{\mathrm{Gen}}{GFR_{K}+\frac{\Delta V}{\Delta t}}=b{\left[\mathrm{Cr}\right]}_{0}$. Then b<1.

Additionally, because of $\frac{\Delta V}{\Delta t}<‐GFR_{K}$ above, $\frac{\mathrm{Gen}}{GFR_{K}+\frac{\Delta V}{\Delta t}}$ must be negative. So, really, b<0.

In Group 2, $\frac{\mathrm{Gen}}{{\left(GFR_{K}+\frac{\Delta V}{\Delta t}\right)}^{2}}=\underset{=b{\left[\mathrm{Cr}\right]}_{0}}{\underbrace{\frac{\mathrm{Gen}}{GFR_{K}+\frac{\Delta V}{\Delta t}}}}\cdot \frac{1}{GFR_{K}+\frac{\Delta V}{\Delta t}}=\frac{b{\left[\mathrm{Cr}\right]}_{0}}{GFR_{K}+\frac{\Delta V}{\Delta t}}$. Substitute these in:

$${\left(\frac{V_{0}}{V_{0}+\frac{\Delta V}{\Delta t}t}\right)}^{\left(1+\frac{GFR_{K}}{\frac{\Delta V}{\Delta t}}\right)}\cdot \frac{1}{\frac{\Delta V}{\Delta t}}\cdot \ln\left(\frac{V_{0}}{V_{0}+\frac{\Delta V}{\Delta t}t}\right)\cdot \left(b{\left[\mathrm{Cr}\right]}_{0}‐{\left[\mathrm{Cr}\right]}_{0}\right)+\frac{b{\left[\mathrm{Cr}\right]}_{0}}{GFR_{K}+\frac{\Delta V}{\Delta t}}\cdot \left[1‐{\left(\frac{V_{0}}{V_{0}+\frac{\Delta V}{\Delta t}t}\right)}^{\left(1+\frac{GFR_{K}}{\frac{\Delta V}{\Delta t}}\right)}\right]<0$$

$${\left(\frac{V_{0}}{V_{0}+\frac{\Delta V}{\Delta t}t}\right)}^{\left(1+\frac{GFR_{K}}{\frac{\Delta V}{\Delta t}}\right)}\cdot \frac{1}{\frac{\Delta V}{\Delta t}}\cdot \ln\left(\frac{V_{0}}{V_{0}+\frac{\Delta V}{\Delta t}t}\right)\cdot \left(b‐1\right)+\underset{\text{Positive}}{\underbrace{\frac{b}{GFR_{K}+\frac{\Delta V}{\Delta t}}}}\cdot \left[1‐{\left(\frac{V_{0}}{V_{0}+\frac{\Delta V}{\Delta t}t}\right)}^{\left(1+\frac{GFR_{K}}{\frac{\Delta V}{\Delta t}}\right)}\right]<0$$

$${\left(\frac{V_{0}}{V_{0}+\frac{\Delta V}{\Delta t}t}\right)}^{\left(1+\frac{GFR_{K}}{\frac{\Delta V}{\Delta t}}\right)}\cdot \frac{GFR_{K}+\frac{\Delta V}{\Delta t}}{\frac{\Delta V}{\Delta t}}\cdot \ln\left(\frac{V_{0}}{V_{0}+\frac{\Delta V}{\Delta t}t}\right)\cdot \frac{b‐1}{b}+1‐{\left(\frac{V_{0}}{V_{0}+\frac{\Delta V}{\Delta t}t}\right)}^{\left(1+\frac{GFR_{K}}{\frac{\Delta V}{\Delta t}}\right)}<0$$

$$e^{\left(1+\frac{GFR_{K}}{\frac{\Delta V}{\Delta t}}\right)\cdot \ln\left(\frac{V_{0}}{V_{0}+\frac{\Delta V}{\Delta t}t}\right)}\cdot \left(1+\frac{GFR_{K}}{\frac{\Delta V}{\Delta t}}\right)\cdot \ln\left(\frac{V_{0}}{V_{0}+\frac{\Delta V}{\Delta t}t}\right)\cdot \frac{b‐1}{b}+1‐e^{\left(1+\frac{GFR_{K}}{\frac{\Delta V}{\Delta t}}\right)\cdot \ln\left(\frac{V_{0}}{V_{0}+\frac{\Delta V}{\Delta t}t}\right)}<0$$

Let $\left(1+\frac{GFR_{K}}{\frac{\Delta V}{\Delta t}}\right)\cdot \ln\left(\frac{V_{0}}{V_{0}+\frac{\Delta V}{\Delta t}t}\right)=x$. Here, $\frac{\Delta V}{\Delta t}<‐GFR_{K}$, which makes $\underset{\text{Positive}}{\underbrace{\left(1+\frac{GFR_{K}}{\frac{\Delta V}{\Delta t}}\right)}}\cdot \underset{\text{Positive}}{\underbrace{\ln\left(\frac{V_{0}}{V_{0}+\frac{\Delta V}{\Delta t}t}\right)}}$ always positive, so x>0.

Substitute in:$e^{x}\cdot x\cdot \frac{b‐1}{b}+1‐e^{x}<0$.

Multiply both sides by $e^{‐x}$: $x\cdot \frac{b‐1}{b}+e^{‐x}‐1<0$.

Let $f\left(x\right)=\frac{b‐1}{b}\cdot x+e^{‐x}‐1$. Is this function always negative when x>0

Differentiate the function: $f^{\prime }\left(x\right)=\frac{b‐1}{b}‐e^{‐x}$.

Because b<0, then $\frac{b‐1}{b}>1$. In contrast, because x>0, then $0<e^{‐x}<1$.

The subtraction yields an $f^{\prime }\left(x\right)$ that is positive, meaning that $f\left(x\right)$ is increasing on x>0.

As x approaches zero from the right, $f\left(x\right)$ must have a minimum of $\lim_{x\to 0^{+}}\frac{b‐1}{b}\cdot x+e^{‐x}‐1=0$.

Thus, $f\left(x\right)$ is always positive, but that contradicts $f\left(x\right)=\frac{b‐1}{b}\cdot x+e^{‐x}‐1$ needing to be negative, so Way #3 does not falsify the hypothesis.

Limit cases:

4. If $\frac{\Delta V}{\Delta t}=0$, then the limit solution is given by Equation (1), shown below:

$$\underset{\frac{\Delta V}{\Delta t}\to 0}{\text{lim}}\frac{\partial {\left[\mathrm{Cr}\right]}_{t}}{\partial GFR_{K}}=e^{\frac{‐GFR_{K}\cdot t}{V_{0}}}\cdot \frac{t}{V_{0}}\cdot \left(\frac{\mathrm{Gen}}{GFR_{K}}‐{\left[\mathrm{Cr}\right]}_{0}\right)‐\left(1‐e^{\frac{‐GFR_{K}\cdot t}{V_{0}}}\right)\cdot \frac{\mathrm{Gen}}{{\left(GFR_{K}\right)}^{2}}$$

Disregarding the trivial case of t=0 that makes $\frac{\partial {\left[\mathrm{Cr}\right]}_{t}}{\partial GFR_{K}}=0$, term by term, the signs have to be

$$\underset{\frac{\Delta V}{\Delta t}\to 0}{\text{lim}}\frac{\partial {\left[\mathrm{Cr}\right]}_{t}}{\partial GFR_{K}}=\underset{\text{Positive}}{\underbrace{e^{\frac{‐GFR_{K}\cdot t}{V_{0}}}}}\cdot \underset{+}{\underbrace{\frac{t}{V_{0}}}}\cdot \underset{\text{can be}\;\pm \;\text{or 0}}{\underbrace{\left(\frac{\mathrm{Gen}}{GFR_{K}}‐{\left[\mathrm{Cr}\right]}_{0}\right)}}‐\underset{\text{Positive}}{\underbrace{\left(1‐e^{\frac{‐GFR_{K}\cdot t}{V_{0}}}\right)}}\cdot \underset{\text{Positive}}{\underbrace{\frac{\mathrm{Gen}}{{\left(GFR_{K}\right)}^{2}}}}$$

(Note: $\frac{\Delta V}{\Delta t}=GFR_{K}=0$ is analyzed later.)

i. If $\frac{\mathrm{Gen}}{GFR_{K}}‐{\left[\mathrm{Cr}\right]}_{0}\leq 0$, then the derivative limit is negative, which supports the hypothesis.

ii. If $\frac{\mathrm{Gen}}{GFR_{K}}‐{\left[\mathrm{Cr}\right]}_{0}>0$, then the derivative limit is potentially positive. Clinically, is it possible that $e^{\frac{‐GFR_{K}\cdot t}{V_{0}}}\cdot \frac{t}{V_{0}}\cdot \left(\frac{\mathrm{Gen}}{GFR_{K}}‐{\left[\mathrm{Cr}\right]}_{0}\right)‐\left(1‐e^{\frac{‐GFR_{K}\cdot t}{V_{0}}}\right)\cdot \frac{\mathrm{Gen}}{{\left(GFR_{K}\right)}^{2}}>0$

Since $\frac{\mathrm{Gen}}{GFR_{K}}>{\left[\mathrm{Cr}\right]}_{0}$, let $\frac{\mathrm{Gen}}{GFR_{K}}=c{\left[\mathrm{Cr}\right]}_{0}$. Then c>1.

Further, $\frac{\mathrm{Gen}}{{\left(GFR_{K}\right)}^{2}}=\underset{=c{\left[\mathrm{Cr}\right]}_{0}}{\underbrace{\frac{\mathrm{Gen}}{GFR_{K}}}}\cdot \frac{1}{GFR_{K}}=\frac{c{\left[\mathrm{Cr}\right]}_{0}}{GFR_{K}}$. Substitute these in:

$$e^{\frac{‐GFR_{K}\cdot t}{V_{0}}}\cdot \frac{t}{V_{0}}\cdot \left(c{\left[\mathrm{Cr}\right]}_{0}‐{\left[\mathrm{Cr}\right]}_{0}\right)‐\left(1‐e^{\frac{‐GFR_{K}\cdot t}{V_{0}}}\right)\cdot \frac{c{\left[\mathrm{Cr}\right]}_{0}}{GFR_{K}}>0$$

$$e^{\frac{‐GFR_{K}\cdot t}{V_{0}}}\cdot \frac{t}{V_{0}}\cdot \left(c‐1\right)‐\left(1‐e^{\frac{‐GFR_{K}\cdot t}{V_{0}}}\right)\cdot \underset{\text{Positive}}{\underbrace{\frac{c}{GFR_{K}}}}>0$$

$$e^{\frac{‐GFR_{K}\cdot t}{V_{0}}}\cdot \frac{GFR_{K}\cdot t}{V_{0}}\cdot \frac{c‐1}{c}‐1+e^{\frac{‐GFR_{K}\cdot t}{V_{0}}}>0$$

Let $\frac{GFR_{K}\cdot t}{V_{0}}=x$. By inspection, x>0 (not considering $GFR_{K}=0$ yet or t=0 at all).

Substitute in:

$$e^{‐x}\cdot x\cdot \frac{c‐1}{c}‐1+e^{‐x}>0$$

Multiply both sides by $e^{x}$: $x\cdot \frac{c‐1}{c}‐e^{x}+1>0$.

Let $f\left(x\right)=\frac{c‐1}{c}\cdot x‐e^{x}+1$. Is this function always positive when x>0

Differentiate the function: $f^{\prime }\left(x\right)=\frac{c‐1}{c}‐e^{x}$.

Because c>1, then $0<\frac{c‐1}{c}<1$. In contrast, because x>0, then $e^{x}>1$.

The subtraction yields an $f^{\prime }\left(x\right)$ that is negative, meaning that $f\left(x\right)$ is decreasing on x>0.

As x approaches zero from the right, $f\left(x\right)$ must have a maximum of $\underset{x\to 0^{+}}{\text{lim}}\frac{c‐1}{c}\cdot x‐e^{x}+1=0$.

Thus, $f\left(x\right)$ is always negative, but that contradicts $f\left(x\right)=\frac{c‐1}{c}\cdot x‐e^{x}+1$ needing to be positive, so the overall derivative's limit as $\frac{\Delta V}{\Delta t}\to 0$ must be negative.

5. If $GFR_{K}=‐\frac{\Delta V}{\Delta t}$, then the limit solution is given by Equation (2), shown below:

$$\underset{GFR_{K}\to ‐\frac{\Delta V}{\Delta t}}{\text{lim}}\frac{\partial {\left[\mathrm{Cr}\right]}_{t}}{\partial GFR_{K}}=‐\frac{1}{\frac{\Delta V}{\Delta t}}\cdot \ln\left(\frac{V_{0}}{V_{0}+\frac{\Delta V}{\Delta t}t}\right)\cdot \left[\frac{\mathrm{Gen}}{2\cdot \frac{\Delta V}{\Delta t}}\cdot \ln\left(\frac{V_{0}}{V_{0}+\frac{\Delta V}{\Delta t}t}\right)‐{\left[\mathrm{Cr}\right]}_{0}\right]$$

Clinically, $GFR_{K}$ is positive, forcing $\frac{\Delta V}{\Delta t}$ to be negative. (Note: $GFR_{K}=\frac{\Delta V}{\Delta t}=0$ is analyzed next.) Then, term by term, the signs have to be:

$‐\underset{\text{Negative}}{\underbrace{\frac{1}{\frac{\Delta V}{\Delta t}}\cdot \ln\left(\frac{V_{0}}{V_{0}+\frac{\Delta V}{\Delta t}t}\right)}}\cdot \left[\underset{\text{Negative}}{\underbrace{\frac{\mathrm{Gen}}{2\cdot \frac{\Delta V}{\Delta t}}\cdot \ln\left(\frac{V_{0}}{V_{0}+\frac{\Delta V}{\Delta t}t}\right)}}‐\underset{\text{Positive}}{\underbrace{{\left[\mathrm{Cr}\right]}_{0}}}\right].$

The negative sign out in front makes the entire derivative limit negative, which supports the hypothesis that $\frac{\partial {\left[\mathrm{Cr}\right]}_{t}}{\partial GFR_{K}}$ is always negative (disregarding the trivial case of t=0).

6. If $GFR_{K}=\frac{\Delta V}{\Delta t}=0$, then the limit solution is given by Equation (4), shown below:

$$\underset{\left(GFR_{K},\frac{\Delta V}{\Delta t}\right)\to \left(0,0\right)}{\text{lim}}\frac{\partial {\left[\mathrm{Cr}\right]}_{t}}{\partial GFR_{K}}=‐\frac{t}{V_{0}}\cdot \left(\frac{Gen\cdot t}{2\cdot V_{0}}+{\left[\mathrm{Cr}\right]}_{0}\right).$$

Disregarding the trivial case of t=0, term by term, the signs have to be $‐\frac{t}{\underset{+}{\underbrace{V_{0}}}}\cdot \left(\frac{Gen\cdot t}{\underset{\text{Positive}}{\underbrace{2\cdot V_{0}}}}+\underset{\text{Positive}}{\underbrace{{\left[\mathrm{Cr}\right]}_{0}}}\right)$.

The negative sign out in front makes the entire derivative limit negative, which supports the hypothesis that $\frac{\partial {\left[\mathrm{Cr}\right]}_{t}}{\partial GFR_{K}}$ is always negative.

Conclusion: All six ways that the partial derivative could be positive were falsified. Therefore, the derivative $\frac{\partial {\left[\mathrm{Cr}\right]}_{t}}{\partial GFR_{K}}$ is always negative (except when it is zero at t=0).

Lastly, the partial derivative of ${\left[\mathrm{Cr}\right]}_{t}$ with respect to $\frac{\Delta V}{\Delta t}$ is derived as follows:

In Equation (2) of the main text, the derivative of $\left[1‐{\left(\frac{V_{0}}{V_{0}+\frac{\Delta V}{\Delta t}t}\right)}^{\left(1+\frac{GFR_{K}}{\frac{\Delta V}{\Delta t}}\right)}\right]$ with respect to $\frac{\Delta V}{\Delta t}$ is $0‐{\left[{\left(\frac{V_{0}}{V_{0}+\frac{\Delta V}{\Delta t}t}\right)}^{\left(1+\frac{GFR_{K}}{\frac{\Delta V}{\Delta t}}\right)}\right]}^{\prime }$.

To calculate the derivative of the exponential, let $y={\left(\frac{V_{0}}{V_{0}+\frac{\Delta V}{\Delta t}t}\right)}^{\left(1+\frac{GFR_{K}}{\frac{\Delta V}{\Delta t}}\right)}$ and use logarithms.

$$\ln y=\ln{\left(\frac{V_{0}}{V_{0}+\frac{\Delta V}{\Delta t}t}\right)}^{\left(1+\frac{GFR_{K}}{\frac{\Delta V}{\Delta t}}\right)}=\left(1+\frac{GFR_{K}}{\frac{\Delta V}{\Delta t}}\right)\cdot \ln\left(\frac{V_{0}}{V_{0}+\frac{\Delta V}{\Delta t}t}\right).$$

Differentiate with the product rule:

$$\frac{1}{y}y^{\prime }=‐\frac{GFR_{K}}{{\left(\frac{\Delta V}{\Delta t}\right)}^{2}}\cdot \ln\left(\frac{V_{0}}{V_{0}+\frac{\Delta V}{\Delta t}t}\right)+\left(1+\frac{GFR_{K}}{\frac{\Delta V}{\Delta t}}\right)\cdot \frac{V_{0}+\frac{\Delta V}{\Delta t}t}{V_{0}}\cdot \frac{‐V_{0}\cdot t}{{\left(V_{0}+\frac{\Delta V}{\Delta t}t\right)}^{2}}.$$

$$\frac{1}{y}y^{\prime }=‐\frac{GFR_{K}}{{\left(\frac{\Delta V}{\Delta t}\right)}^{2}}\cdot \ln\left(\frac{V_{0}}{V_{0}+\frac{\Delta V}{\Delta t}t}\right)‐\left(1+\frac{GFR_{K}}{\frac{\Delta V}{\Delta t}}\right)\cdot \frac{t}{V_{0}+\frac{\Delta V}{\Delta t}t}.$$

$$y^{\prime }=‐{\left(\frac{V_{0}}{V_{0}+\frac{\Delta V}{\Delta t}t}\right)}^{\left(1+\frac{GFR_{K}}{\frac{\Delta V}{\Delta t}}\right)}\cdot \left[\frac{GFR_{K}}{{\left(\frac{\Delta V}{\Delta t}\right)}^{2}}\cdot \ln\left(\frac{V_{0}}{V_{0}+\frac{\Delta V}{\Delta t}t}\right)+\left(1+\frac{GFR_{K}}{\frac{\Delta V}{\Delta t}}\right)\cdot \frac{t}{V_{0}+\frac{\Delta V}{\Delta t}t}\right].$$

Thus,

$${\left[1‐{\left(\frac{V_{0}}{V_{0}+\frac{\Delta V}{\Delta t}t}\right)}^{\left(1+\frac{GFR_{K}}{\frac{\Delta V}{\Delta t}}\right)}\right]}^{\prime }={\left(\frac{V_{0}}{V_{0}+\frac{\Delta V}{\Delta t}t}\right)}^{\left(1+\frac{GFR_{K}}{\frac{\Delta V}{\Delta t}}\right)}\cdot \left[\frac{GFR_{K}}{{\left(\frac{\Delta V}{\Delta t}\right)}^{2}}\cdot \ln\left(\frac{V_{0}}{V_{0}+\frac{\Delta V}{\Delta t}t}\right)+\left(1+\frac{GFR_{K}}{\frac{\Delta V}{\Delta t}}\right)\cdot \frac{t}{V_{0}+\frac{\Delta V}{\Delta t}t}\right].$$

Next, find the derivative of $\left(\frac{\mathrm{Gen}}{GFR_{K}+\frac{\Delta V}{\Delta t}}‐{\left[\mathrm{Cr}\right]}_{0}\right)$ with respect to $\frac{\Delta V}{\Delta t}$:

$${\left(\frac{\mathrm{Gen}}{GFR_{K}+\frac{\Delta V}{\Delta t}}‐{\left[\mathrm{Cr}\right]}_{0}\right)}^{\prime }=‐\frac{\mathrm{Gen}}{{\left(GFR_{K}+\frac{\Delta V}{\Delta t}\right)}^{2}}.$$

Using the two intermediate derivatives for the product rule applied to Equation (2) of the main text, we find that $\frac{\partial {\left[\mathrm{Cr}\right]}_{t}}{\partial \frac{\Delta V}{\Delta t}}$ is:

$$\begin{array}{cc} \frac{\partial {\left[\mathrm{Cr}\right]}_{t}}{\partial \frac{\Delta V}{\Delta t}} & ={\left(\frac{V_{0}}{V_{0}+\frac{\Delta V}{\Delta t}t}\right)}^{\left(1+\frac{GFR_{K}}{\frac{\Delta V}{\Delta t}}\right)}\cdot \left[\frac{GFR_{K}}{{\left(\frac{\Delta V}{\Delta t}\right)}^{2}}\cdot \ln\left(\frac{V_{0}}{V_{0}+\frac{\Delta V}{\Delta t}t}\right)+\left(1+\frac{GFR_{K}}{\frac{\Delta V}{\Delta t}}\right)\cdot \frac{t}{V_{0}+\frac{\Delta V}{\Delta t}t}\right]\\ & \;\times \left(\frac{\mathrm{Gen}}{GFR_{K}+\frac{\Delta V}{\Delta t}}‐{\left[\mathrm{Cr}\right]}_{0}\right)+\left[1‐{\left(\frac{V_{0}}{V_{0}+\frac{\Delta V}{\Delta t}t}\right)}^{\left(1+\frac{GFR_{K}}{\frac{\Delta V}{\Delta t}}\right)}\right]\cdot \frac{‐Gen}{{\left(GFR_{K}+\frac{\Delta V}{\Delta t}\right)}^{2}}. \end{array}$$
